# Gender differences in diet-induced steatotic disease in Cyp2b-null mice

**DOI:** 10.1371/journal.pone.0229896

**Published:** 2020-03-10

**Authors:** Melissa M. Heintz, Rebecca McRee, Ramiya Kumar, William S. Baldwin

**Affiliations:** 1 Environmental Toxicology Program, Clemson University, Clemson, SC, United States of America; 2 Biological Sciences, Clemson University, Clemson, SC, United States of America; Medizinische Fakultat der RWTH Aachen, GERMANY

## Abstract

Nonalcoholic fatty liver disease (NAFLD) is the most common liver disease; however, progression to nonalcoholic steatohepatitis (NASH) is associated with most adverse outcomes. CYP2B metabolizes multiple xeno- and endobiotics, and male Cyp2b-null mice are diet-induced obese (DIO) with increased NAFLD. However, the DIO study was not performed long enough to assess progression to NASH. Therefore, to assess the role of Cyp2b in fatty liver disease progression from NAFLD to NASH, we treated wildtype (WT) and Cyp2b-null mice with a normal diet (ND) or choline-deficient, L-amino acid-defined high fat diet (CDAHFD) for 8 weeks and determined metabolic and molecular changes. CDAHFD-fed WT female mice gained more weight and had greater liver and white adipose tissue mass than their Cyp2b-null counterparts; males experienced diet-induced weight loss regardless of genotype. Serum biomarkers of liver injury increased in both CDAHFD-fed female and male mice; however CDAHFD-fed Cyp2b-null females exhibited significantly lower serum ALT, AST, and ASP concentrations compared to WT mice, indicating Cyp2b-null females were protected from liver injury. In both genders, hierarchical clustering of RNA-seq data demonstrates several gene ontologies responded differently in CDAHFD-fed Cyp2b-null mice compared to WT mice (lipid metabolism > fibrosis > inflammation). Oil Red O staining and direct triglycerides measurements confirmed that CDAHFD-fed Cyp2b-null females were protected from NAFLD. CDAHFD-fed Cyp2b-null mice showed equivocal changes in fibrosis with transcriptomic and serum markers suggesting less inflammation due to glucocorticoid-mediated repression of immune responses. In contrast to females, CDAHFD-fed Cyp2b-null males had higher triglyceride levels. Results indicate that female Cyp2b-null mice are protected from NAFLD while male Cyp2b-null mice are more susceptible to NAFLD, with few significant changes in NASH development. This study confirms that increased NAFLD development does not necessarily lead to progressive NASH. Furthermore, it indicates a role for Cyp2b in fatty liver disease that differs based on gender.

## Introduction

Liver disease often progresses from nonalcoholic fatty liver disease (NAFLD) to more severe diseases such as nonalcoholic steatohepatitis (NASH), fibrosis including cirrhosis, and liver cancer [1]. NAFLD is the most common liver disease and increasing in prevalence, with 10–30% of U.S. citizens and 25% of people worldwide diagnosed [2]. NAFLD is closely linked to obesity [3] and is the result of the hepatic manifestation of the metabolic syndrome [4]. NAFLD is defined as the presence of ≥ 5% hepatic steatosis in the absence of other liver diseases [2], yet less than 25% of patients with NAFLD develop NASH [5]. In some cases the hepatic intracellular accumulation of lipids in fatty liver disease can develop into NASH as inflammation, injury and fibrosis progress due to anti-lipotoxic protection failure [5]. NASH is recognized as one of the primary causes of cirrhosis in adults and is currently the second most prominent cause for liver transplants in the United States [6]. Studies have shown that steatosis may not affect NASH development [7]; therefore, the pathogenesis of NASH remains controversial. However, fibrosis progression occurs in most patients with NASH and cirrhosis is the main histological feature associated with mortality of NASH patients [8].

Regardless of how these diseases progress, dietary factors are the primary source for both NAFLD and NASH [7]. A rise in consumption of foods high in polyunsaturated fatty acids (PUFAs) including vegetable and soybean oil parallels the increase in obesity in the United States and worldwide since the 1970s [9]. During inflammation, PUFAs found in hepatic membranes, are released by phospholipase A2. These free PUFAs are then oxidized by cyclooxygenase, lipoxygenase, or cytochrome P450s (CYPs) to form physiologically significant metabolites. CYP-derived epoxides include the epoxyeicosatrienoic acids (EETs) produced from the metabolism of arachidonic acid (AA) [10, 11]; the epoxyoctadecenoic acids (EpOMEs) from linoleic acid; the epoxyoctadecadienoic acids from α-linolenic acid; the epoxyeicosatetraenoic acids from eicosapentaenoic acid (EPA); and the epoxydocosapentaenoic acids from docosahexaenoic acid (DHA) [12, 13]. Some PUFAs regulate CYP activity, such as DHA which binds to the retinoid X receptor (RXR) [14] and prevents constitutive androstane receptor (CAR) translocation to the nucleus and subsequent transcription of Cyp2b and other CAR biomarker proteins [15]. Conversely, linoleic acid induces Cyp2b expression via activation of CAR [16].

In the liver, CAR regulates both human and murine Cyp2b genes [17–19]. *Cyp2b9*, *Cyp2b10*, and *Cyp2b13* make up the primary hepatic Cyp2b genes in mice, and *CYP2B6*, the only *CYP2B* member in humans is highly expressed in the liver [20]. Several studies indicate a role for Cyp2b in metabolizing fatty acids. Murine Cyp2b19, found in keratinocytes, metabolizes arachidonic acid to 11,12- and 14,15-EET that are important for a functional epidermis [21]. However, human CYP2B6 does not produce significant arachidonic acid metabolites [22]. Interestingly, anandamide, an arachidonic acid-derived endogenous cannabinoid, is metabolized by CYP2B6 into four EET metabolites [23], including 5,6-epoxyeicosatetraenoic acid ethanolamide, which has been found to be a potent agonist of the peripheral cannabinoid receptor, CB2 [24].

Finn et al [16] found that loss of all hepatic CYP activity in the hepatic P450 oxidoreductase (POR)-null mouse model led to hepatic steatosis and the induction of Cyp2b10 through the activation of CAR, a putative anti-obesity receptor [18, 25]. The authors hypothesized that Cyp2b and to a lesser extent Cyp3a enzymes play a backup role in the metabolism and protection from the build-up of fatty acids in the liver [16]. Additional studies show CAR’s important role in recognizing hepatic lipids via fat metabolism regulation [26], caloric restriction [27], obesity [28], and bile acid homeostasis [29]. Furthermore, several studies performed in mice demonstrated that *Cyp2b9* exhibited the highest increased expression by RNAseq following a high fat diet (HFD) [30, 31].

In addition, RNAi-based Cyp2b-knockdown mice on an FVB/NJ background display increased adiposity and body weight with age as well as a decreased ability to eliminate PUFA-rich corn oil, primarily in males [32]. In our recently published research, C57Bl/6J (B6)-Cyp2b9/10/13-null (Cyp2b-null) mice lacking the predominantly hepatic Cyp2b members, *Cyp2b9*, *Cyp2b10*, and *Cyp2b13*, are diet-induced obese in males with moderate increases in steatosis. Interestingly, the Cyp2b-null male mice developed some steatosis regardless of diet; however, they showed very little hepatic inflammation, which is unusual, suggesting Cyp2b-null mice may be protected from developing NASH [33]. This study was only carried out for 10 weeks and new research indicates that B6 mice fed a high fructose + 42% Kcal HFD for up to 22-weeks failed to form NASH following NAFLD development. Therefore, the previous study was not equipped to investigate NASH development in Cyp2b-null mice. Based on the lack of inflammation in HFD-fed Cyp2b-null mice, we predict that diet-induced NASH will increase hepatic triglycerides in Cyp2b-null mice. This increase in triglycerides will provide protection from liver inflammation and injury compared to their WT counterparts, as free fatty acids and their metabolites are more lipotoxic than inert triglycerides [34].

We used our previously developed Cyp2b-null mouse model [33, 35] to test whether a lack of Cyp2b plays a role in the progression of liver disease. Cyp2b-null and WT mice (n = 9) were fed either a choline-deficient, L-amino acid-defined, high fat diet (CDAHFD) or normal diet (ND) for 8 weeks. The CDAHFD is similar to a methionine-choline deficient (MCD) diet model for the determination of NAFLD/NASH. However, the MCD diet exhibits severe body weight loss which makes long term studies difficult [36]. CDAHFD-fed mice do not lose weight, but instead gradually gain weight and develop steatohepatitis and fibrosis more rapidly [37]. Both physiological and biochemical changes, including differential gene expression via RNA sequencing were examined in treated WT and Cyp2b-null mice. Results from this study show that a lack of Cyp2b causes gender-based differences in response to diet-induced NASH treatment.

## Materials and methods

### Choline-deficient, L-amino acid-defined high-fat diet (CDAHFD)

Animal care and associated procedures were approved by Clemson University’s Institutional Animal Care and Use committee. Cyp2b9/10/13-null (Cyp2b-null) mice were developed using CRISPR/Cas9 technology on the C57Bl/6J (B6) background mice as previously described [33, 35]. Wildtype (WT) B6 mice were purchased from The Jackson Laboratory (Bar Harbor, ME, USA) at 6 weeks of age and were acclimated for 4 weeks prior to treatment. WT and Cyp2b-null male and female (10 weeks old) mice were divided into groups (n = 9) and fed either a normal chow diet (ND; Harlan, 3.1 Kcal/g: 18.6% protein, 6.2% fat, 44.2% carbohydrates; Madison, WI USA) or a CDAHFD (Research Diets, 5.2 Kcal/g: 18% protein, 62% fat, 20% carbohydrates, 0.1% methionine; New Brunswick, NJ, USA) for 8 weeks [37]. Weight gain was monitored weekly and feed consumption was measured every other day. Fasting blood glucose levels were determined during weeks 4 and 6. Glucose tolerance tests (GTT) were performed during week 6. At the end of the study, mice were anesthetized and blood collected by heart puncture prior to euthanasia. Liver, kidney, white adipose tissue (WAT), and brown adipose tissue (BAT) were excised and weighed. All tissues were immediately snap frozen with liquid nitrogen and stored at -80°C or placed in 10% formalin (Fisher, Fairlawn, NJ USA) for further studies. A timeline of procedures is provided (**S1 Fig**).

### Fasting blood glucose and glucose tolerance tests

During weeks 4 and 6, mice were fasted for 4 hours and fasting blood glucose was determined using an Alphatrak 2 (AlphaTRAK, Chicago, IL USA) blood glucose meter following tail bleed. During week 6, glucose tolerance was determined following an intraperitoneal injection of 1g/kg of their body weight of D-glucose (Sigma Ultra, St. Louis, MO USA) with blood glucose readings every 20 min for the first hour and every 30 min for the second hour as described previously [33, 38].

### Serum biomarker panel

Blood samples were collected by heart puncture and incubated at room temperature for 30 min followed by centrifugation at 6000 rpm for 10 min. Serum from each sample was transferred into a fresh tube and aliquots shipped on dry ice to Baylor College of Medicine’s Comparative Pathology Laboratory (Houston, TX USA) for determination of tissuse damage serum marker concentrations including alanine aminotransferase (ALT), aspartate aminotransferase (AST), alkaline phosphatase (ALP), and creatine kinase (CK), and lactate dehydrogenase (LDH). In addition, serum glucose, cholesterol, triglycerides, high density lipoprotein (HDL), low density lipoprotein (LDL), very low density lipoprotein (VLDL), phosphorus, calcium, and direct, indirect, and total bilirubin were quantified. Serum parameters were determined using a Beckman Coulter AU480 chemistry analyzer (Atlanta, GA, USA) and the appropriate Beckman Coulter biochemical kits according to the manufacturer’s instructions.

### Serum concentrations of leptin, adiponectin, corticosterone, β-hydroxybutyrate, and C-reactive protein

Serum leptin, adiponectin and β-hydroxybutyrate concentrations were determined using EIA or colorimetric kits from Cayman Chemical (Ann Arbor, MI), and serum corticosterone and C-reactive protein (CRP) from Abcam (Cambridge, MA USA) according to the manufacturer’s instructions.

### Liver triglyceride, cholesterol, and hydroxyproline

Liver triglycerides, cholesterol and hydroxyproline were extracted and quantified as described previously [39] using colorimetric kits from Cayman Chemical (triglycerides only) and Abcam according to the manufacturer’s instructions.

### Histopathological analysis

During necropsy, clean liver slices were placed in 10% formalin (Fisher) or snap frozen in liquid nitrogen for staining. Slices placed in 10% formalin were stained with Hematoxylin and Eosin (H&E) or Masson’s trichrome at Colorado Histoprep (Fort Collins, CO USA). Slices snap frozen in liquid nitrogen were stained with Oil Red O at Baylor College of Medicine’s Comparative Pathology Laboratory using standard protocols [26]. The liver lipid droplets stained by Oil Red O were quantified by total area of each sample imaged (400x magnification) using ImageJ Fiji [40]. In Fiji, the scale bar was set to equal 0.05 mm and threshold color to red, with red pass selected and green/blue pass deselected. Red coverage of particles was set to 130–255 and size (mm^2^) equals 0.00001-infinity for all images measured.

### Immunoblots

Nuclear protein extracts were prepared by homogenizing frozen livers followed by differential centrifugation using a Nuclear Protein Extraction kit (Cayman Chemical). Protein concentrations were determined using Bradford reagent (Bio-Rad). Immunoblots were performed using 30 μg of nuclear protein separated on a 12% SDS-polyacrylamide gel (BioRad) and then protein was transferred onto a 0.2μm polyvinylidene difluoride (PVDF) membrane. Specific proteins were recognized using polyclonal antibodies to Proliferating Cell Nuclear Antigen (PCNA) (ThermoFisher, Waltham, MA) and β-actin (Sigma Aldrich, St. Louis MO USA) as the reference protein. Chemiluminescent immunoblot detection was done using alkaline phosphatase conjugated secondary antibodies where anti-rabbit IgG (Immunostar, Bio-Rad) was used to visualize PCNA and anti-mouse IgG (Immunostar, Bio-Rad) was used to visualize β-actin. Protein bands were quantified by densitometry (Image Lab 6.0.1, BioRad, Hercules, CA). Relative density is shown as the average of two samples using β-actin as the reference gene. Data are presented as relative mean of WT ND compared to each treatment group.

### RNA sequencing (RNAseq)

Liver samples were stored in RNAlater Stabilization Solution (Invitrogen, Carlsbad, CA USA) at -80°C. Total RNA was extracted from mouse livers of each treatment group using TRIzol (Ambion, Carlsbad, CA USA) and quantified on a Qubit 2.0 Fluorometer. RNA integrity number (RIN) was determined with an Agilent 2100 Bioanalyzer (place) to assess RNA quality, and samples with a RIN > 8.0 were determined to be of high quality and used for next generation sequencing. Libraries were prepared using NEB Next Ultra RNA Library Prep kit. Samples were sequenced to an average sequencing depth of 20,000,000 read pairs with a 2x150 paired-end module using a NovaSeq 6000. Quality metrics were checked using FastQC on all samples sequenced, and Trimmomatic was used to trim low quality bases. Trimmed reads were aligned to the *Mus musculus* reference genome (GCF_000001635.25_GRCm38.p6) using GSNAP, and 99.8% of the trimmed reads aligned. Subread feature counts software found reads that aligned with known genes. Raw read counts and EdgeR were used to determine differential gene expression [41]. Series GSE137449 containing the RNAseq data has been uploaded to the Gene Expression Omnibus (GEO).

Differential gene expression by multiple comparisons for all treatment groups was determined by EdgeR. Results were filtered for p- and FDR values < 0.05. Normalized counts from the remaining genes post-filtering were compared between groups by Student’s t-tests to determine significantly different (p < 0.05) expressed genes. Heatmap hierarchical cluster analysis by Euclidean distance using Ward’s method was performed with Heatmap.2 in R (https://www.r-project.org/) using significant differentially expressed genes with a log_2_FC > 1.0 between CDAHFD-fed Cyp2b-null and CDAHFD-fed WT groups. GOSeq, a gene ontology (GO) term enrichment analysis program was used to adjust for gene length and expression bias, and create GO term lists for the significant differentially expressed genes between CDAHFD-fed groups in female and male mice [42]. Significantly (p < 0.05) enriched GO terms were visualized in Revigo, which reduces enriched term redundancy and displays the remaining GO terms in a scatterplot [43]. Differentially expressed genes acoss treatment groups were annotated using PANTHER (http://www.pantherdb.org/) and InterPro [44], and differentially expressed genes between CDAHFD-fed Cyp2b-null and CDAHFD-fed WT mice were entered into KEGG Mapper [45] to determine and visualize biochemical pathways perturbed by diet-induced NASH and/or a lack of Cyp2b enzymes [33].

### Quantitative real-time polymerase chain reaction (qPCR)

cDNA was prepared from RNA with MMLV reverse transcriptase, a dNTP mixture, and random hexamers (Promega Corporation, Madison WI). qPCR was used to quantify changes in gene expression using previously published primers [46] and newly developed primers for genes involved in inflammation, fibrosis, and lipid metabolism (S1 Table). PCR efficiency was determined based on a standard curve prepared using a sample mixture containing all the cDNA samples diluted at 1:1, 1:4, 1:16, 1:64, 1:256 and 1:1024 in triplicate with 0.25X RT_2_ SybrGreen (Qiagen Frederick, MD USA) on a CFX 96-well Real-Time PCR detection system (Bio-Rad). 18S was used as the reference gene and Muller’s inverted equation was used to quantify differences in gene expression [47, 48].

### Tests of statistical significance

Data are presented as mean ± SEM (n = 8–9). Statistical analyses were performed by one-way ANOVA followed by Fisher’s LSD as the post-hoc using Graphpad Prism version 7 (GraphPad Software, San Diego, CA). A p-value < 0.05 was considered statistically significant.

## Results

### Changes in body mass following a CDAHFD

**Fig 1:**
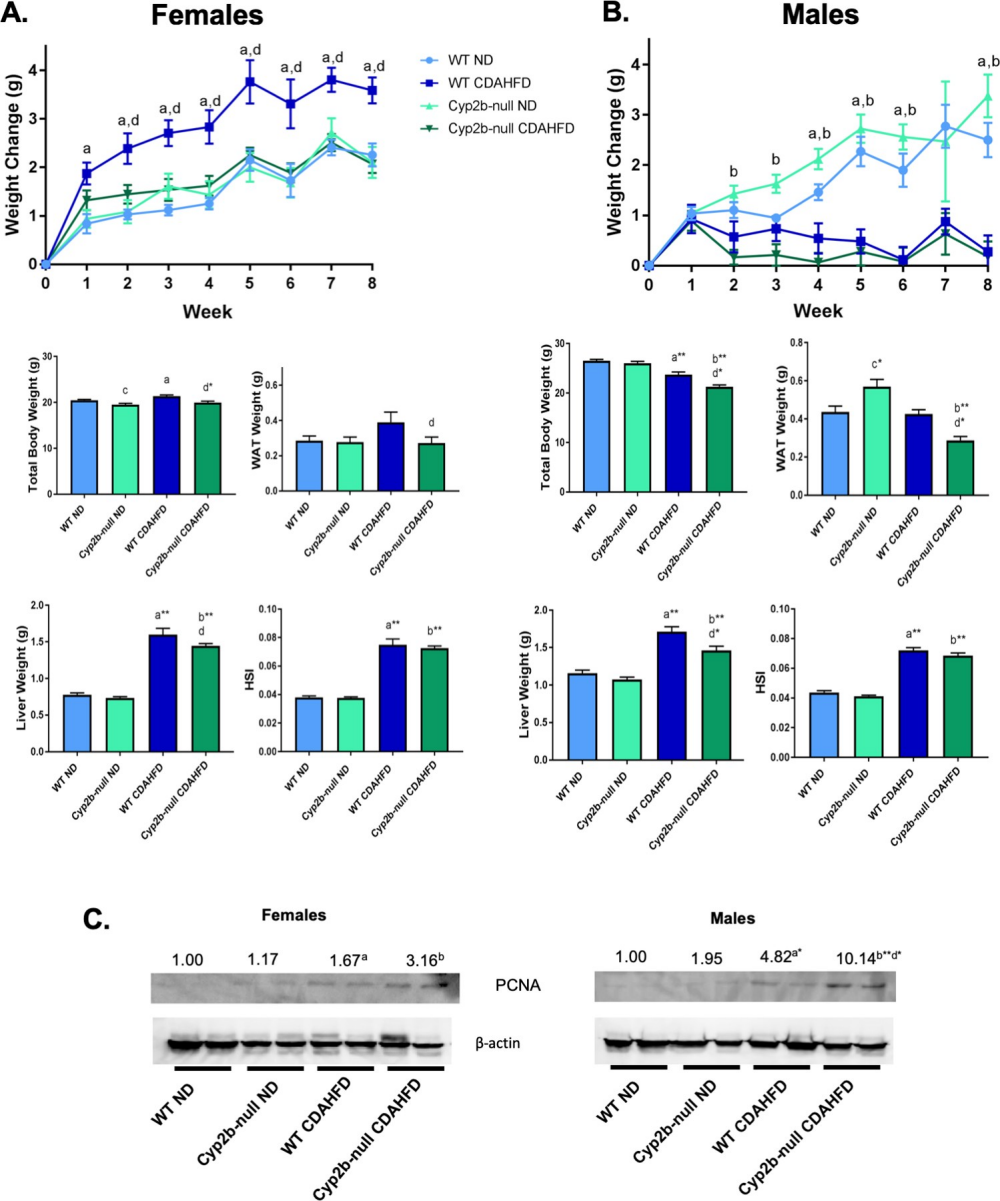
Changes in body weight and organ weight over the 8 weeks of diet-induced NASH treatment. Body and select organ weights of female (A) and male (B) WT and Cyp2b-null mice were monitored during the 8 weeks of treatment. (C) Immunoblots of PCNA in female and male mice with β-actin as the reference gene. Data are presented as mean ± SEM. Statistical significance was determined by one-way ANOVA followed by Fisher’s LSD as the post-hoc test (n = 9; n = 2 for immunoblots). An ‘a’ indicates ND-fed WT different than CDAHFD-fed WT, ‘b’ indicates ND-fed Cyp2b-null different than CDAHFD-fed Cyp2b-null, ‘c’ indicates ND-fed WT different than ND-fed Cyp2b-null, ‘d’ indicates CDAHFD-fed WT different than CDAHFD-fed Cyp2b-null.

CDAHFD-fed WT female mice gained the most weight; significantly more than CDAHFD-fed Cyp2b-null female mice after only two weeks and this trend continued throughout the study. This indicates the lack of Cyp2b enzymes had a substantial repressive effect on CDAHFD-mediated weight gain in females (**Fig 1A**). In contrast, CDAHFD-fed male mice weighed significantly less than their normal diet counterparts, indicating that diet was the primary modifier of weight in males, not genotype (**Fig 1B**). Previous research indicated one of the benefits of a CDAHFD is no weight loss [37]; nevertheless, there was little weight gain in male mice fed a CDAHFD (**Fig 1B**). Mice fed a CDAHFD consumed more calories compared to their ND counterparts; however, genotype did not effect calorie intake (**S2 Fig**). Therefore, the differences in weight between CDAHFD-fed WT and CDAHFD-fed Cyp2b-null female mice cannot be explained by alterations in caloric uptake.

The weight gain in CDAHFD-fed WT female mice was in part attributed to greater liver and white adipose tissue (WAT) mass than CDAHFD-fed Cyp2b-null mice (**Fig 1A**). The lower liver mass in Cyp2b-null females following a CDAHFD suggests a protective effect of Cyp2b loss on liver proliferation or the development of fatty liver disease; however, liver weight decreased in relative proportion to body weight based on the hepatosomatic index (HSI). In contrast to females, ND-fed Cyp2b-null male mice accumulated more white adipose tissue than all other groups (**Fig 1B**). The increase in white adipose tissue in ND-fed Cyp2b-null males is consistent with previous results in Cyp2b-null mice [33]. Similar to females, CDAHFD-fed Cyp2b-null male livers and WAT weighed less than CDAHFD-fed WT mice; however, liver weight was altered in proportion to body weight (HSI) (**Fig 1B**). To determine if liver weight or HSI was more indicative of cell proliferation, we examined proliferative cell nuclear antigen (PCNA) protein levels by immunoblotting (**Fig 1C**). PCNA levels increased in response to a CDAHFD. This response was significantly greater in the male CDAHFD-fed Cyp2b-null mice than the male CDAHFD-fed WT mice, indicating that at least in Cyp2b-null males liver weight was likely increased due to greater proliferation.

### Serum glucose and glucose tolerance in response to a CDAHFD are gender-dependent

**Fig 2:**
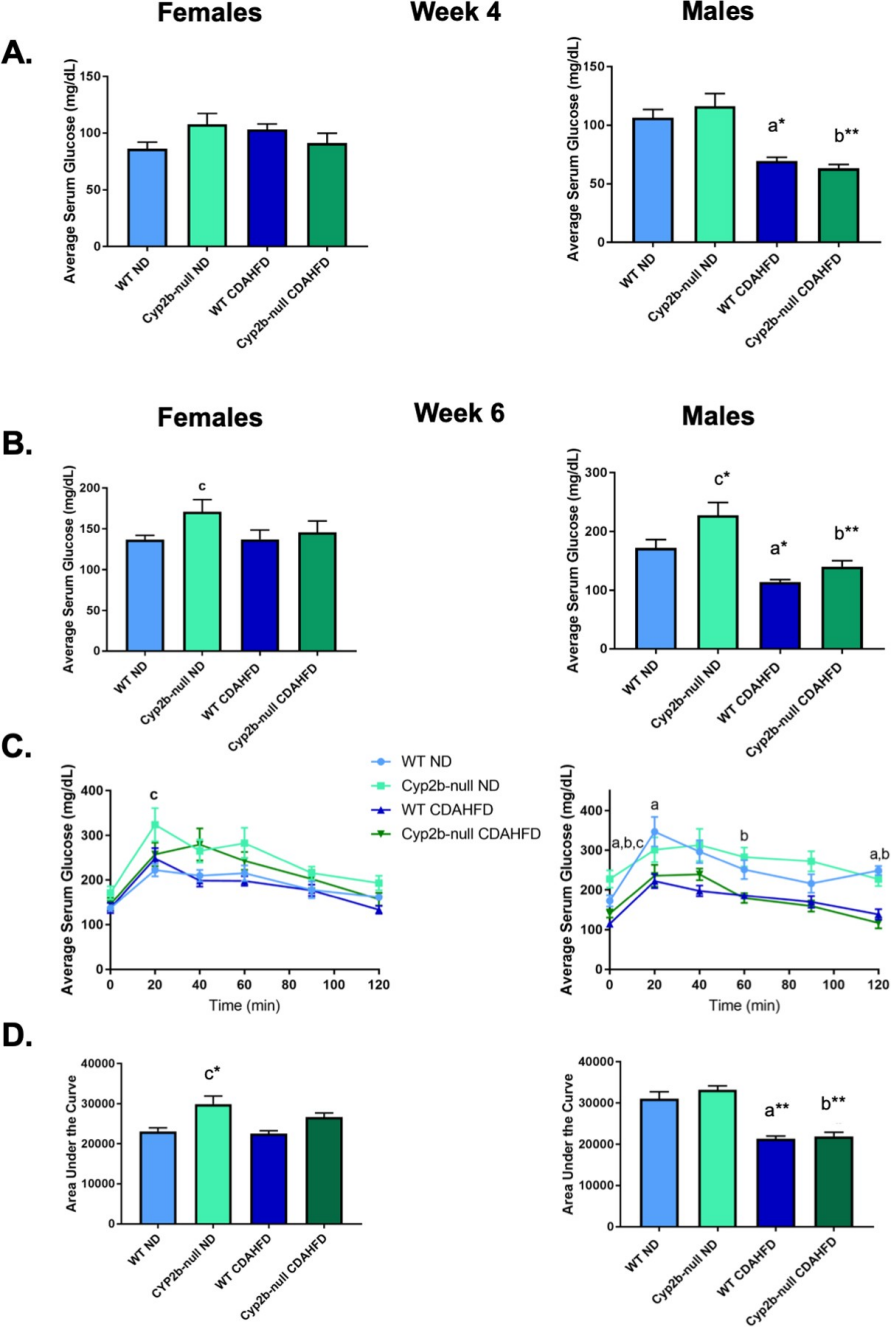
Gender-dependent differences in serum glucose and glucose tolerance in response to CDAHFD. Fasting blood glucose levels were measured during weeks 4 (A) and 6 (B) in female and male mice. During week 6 glucose tolerance tests were performed on all treatment groups (C) and glucose measured at 20, 40, 60, 90, and 120 minutes after the glucose challenge. Results are also represented as area under the curve (D). Data are presented as mean serum glucose ± SEM. Statistical significance was determined by one-way ANOVA followed by Fisher’s LSD as the post-hoc test (n = 9). An ‘a’ indicates ND-fed WT different than CDAHFD-fed WT, ‘b’ indicates ND-fed Cyp2b-null different than CDAHFD-fed Cyp2b-null, ‘c’ indicates ND-fed WT different than ND-fed Cyp2b-null. No asterisk indicates a p-value < 0.05, * indicates a p-value < 0.01, and ** indicates a p-value < 0.0001.

Fasting serum glucose levels measured during weeks 4 and 6 were significantly decreased by the CDAHFD diet in males, but not females (**Fig 2A and 2B**). Fasting serum glucose was not effected by the combination of diet and genotype in either sex. However, fasting serum glucose was increased in ND-fed Cyp2b-null female (25%) and male (32%) mice at week 6 (**Fig 2B**), consistent with previous findings [33]. Glucose tolerance tests (GTT) were performed to determine if Cyp2b-null mice have reduced ability to respond to glucose, a biomarker of metabolic disease. ND-fed Cyp2b-null female mice were slower to clear serum glucose compared to ND-fed WT females; however, there were no differences attributed to diet or a combination of genotype and diet (**Fig 2C and 2D**). In contrast to females, ND-fed Cyp2b-null males showed no difference in glucose tolerance compared to WT mice; CDAHFD-fed males exhibited a significantly faster response to glucose (better glucose tolerance) than ND-fed males regardless of genotype (**Fig 2C and 2D**). In general, poor glucose tolerance was genotype dependent in females and weight or diet dependent in males.

### Serum biomarkers indicate Cyp2b-loss is protective against liver injury in females

**Fig 3:**
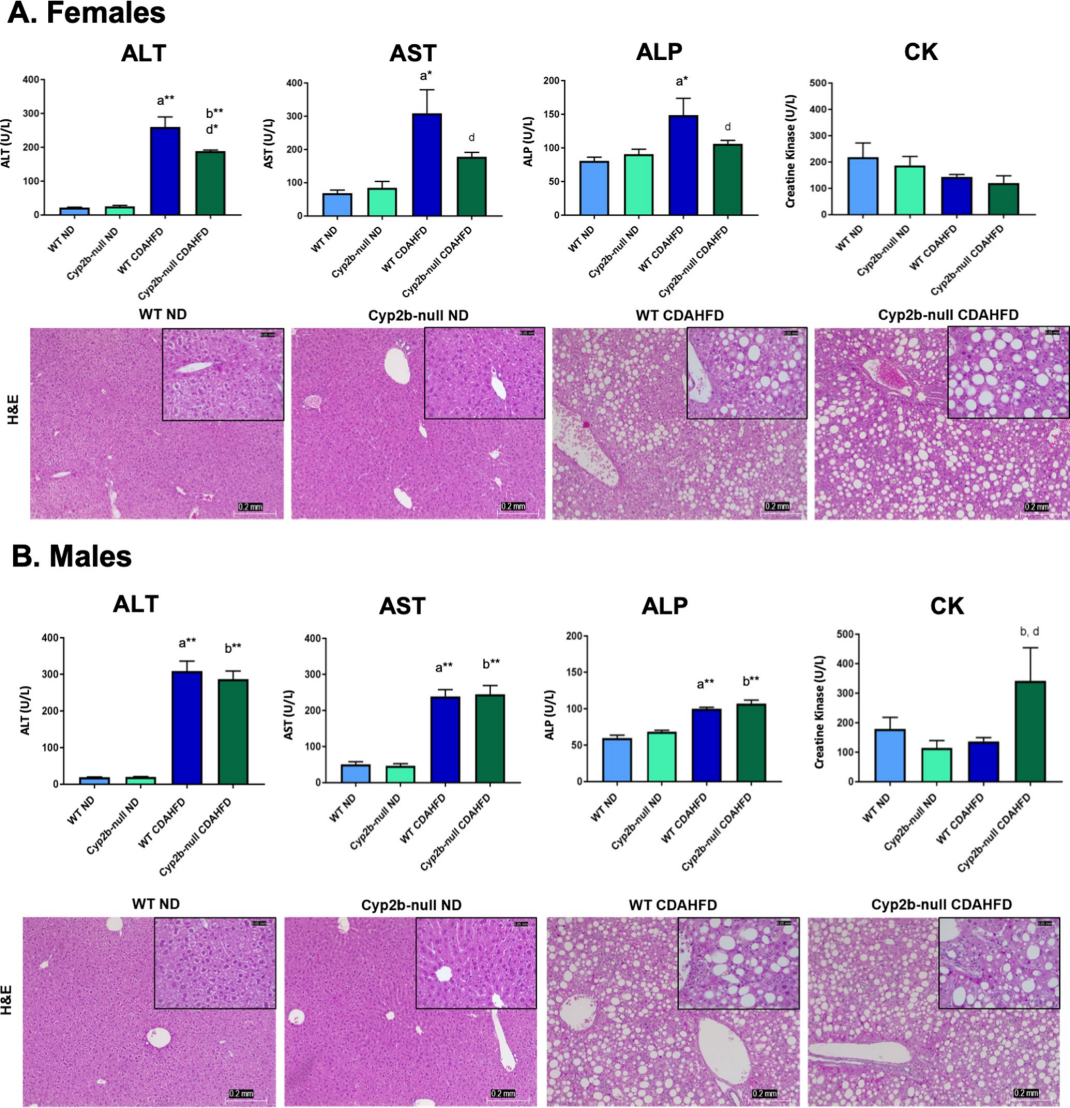
Biomarkers of liver tissue damage in ND and CDAHFD-fed WT and Cyp2b-null mice. Serum ALT, AST, ALP, and CK concentrations were measured, and histopathological changes were evaluated by H&E staining of liver tissues from female (A) and male (B) mice. Images were taken at 100x (0.2 mm) and 400x (0.05 mm) magnification. Data are presented as mean ± SEM. Statistical significance was determined by one-way ANOVA followed by Fisher’s LSD as the post-hoc test (n = 5). An ‘a’ indicates ND-fed WT different than CDAHFD-fed WT, ‘b’ indicates ND-fed Cyp2b-null different than CDAHFD-fed Cyp2b-null, ‘c’ indicates ND-fed WT different than ND-fed Cyp2b-null, ‘d’ indicates CDAHFD-fed WT different than CDAHFD-fed Cyp2b-null. No asterisk indicates a p-value < 0.05, * indicates a p-value < 0.01, and ** indicates a p-value < 0.0001.

Common serum biomarkers of liver injury such as ALT, AST, and ALP increased in female mice fed a CDAHFD; however, CDAHFD-fed Cyp2b-null females exhibited significantly lower serum levels of these enzymes (28%, 42%, 29% lower respectively) compared to their WT counterparts, indicating Cyp2b-null female mice are protected from diet-induced NASH (**Fig 3A**). H&E staining revealed cytoplasmic vacuolization, a marker of cell death [49] caused by the CDAHFD; however, no significant differences in pathology were observed between WT and Cyp2b-null mice (Fig 3A and 3B). Male mice fed a CDAHFD showed increased levels of serum ALT, AST, and ALP regardless of genotype (**Fig 3B**). CDAHFD-fed Cyp2b-null males had higher levels of creatine kinase (CK) (1.5-fold) compared to all other treatment groups including their WT counterparts, suggesting that Cyp2b-null males are more susceptible to CDAHFD-induced damage in other tissues such as cardiac and skeletal muscle [50], the opposite of females (**Fig 3B**).

### Perturbed gene expression in Cyp2b-null mice fed a CDAHFD

**Fig 4:**
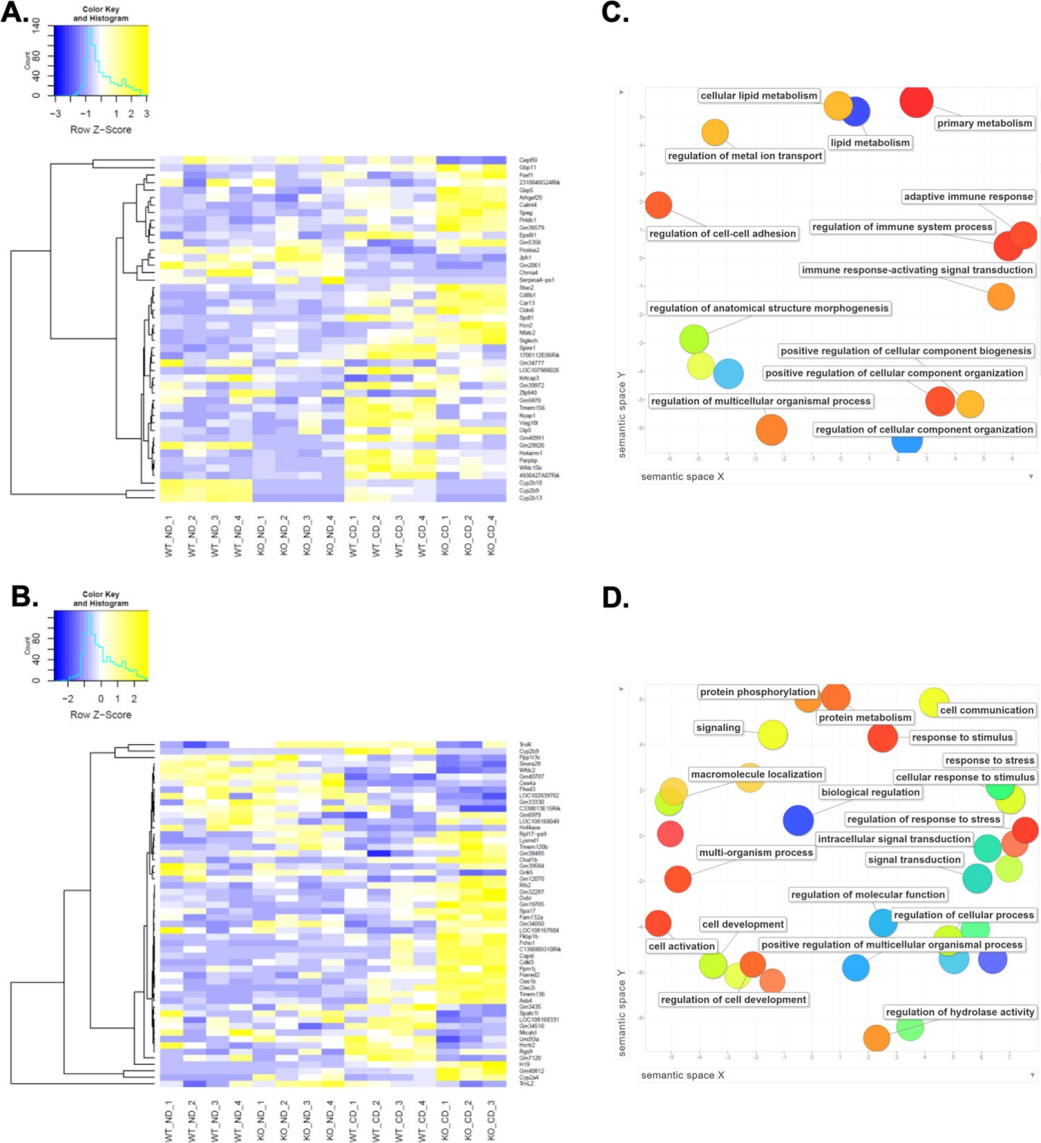
RNAseq demonstrates changes in gene expression in livers of CDAHFD-fed Cyp2b-null mice. Heat maps showing log2-transformed, Z-score scaled RNA-seq expression of significant differentially expressed genes (log_2_FC > 1.0) between CDAHFD-fed Cyp2b-null and CDAHFD-fed WT groups in female (A) and male (B) mice. Yellow and blue color intensity indicate gene up- or down-regulation, respectively. Dendrogram clustering on the y-axis groups genes by expression profile across samples. GO term enrichment analysis summary using Revigo [43] for up-regulated GO terms in CDAHFD-fed Cyp2b-null female (C) and male (D) mice compared to CDAHFD-fed WT mice. Each scatterplot contains enriched GO terms from the biological process class that remain after term redundancy is reduced and are displayed in a two-dimensional space where semantically similar GO terms are positioned closer together within the plot. Each circle represents an enriched GO term; the cooler the color of a term, the greater signficance (p < 0.05) of that term with measured changes in gene expression. Circle size indicates the frequency of the GO term in the underlying GO database, i.e. circles of more general terms are larger.

RNA-seq was performed on liver samples from all treatment groups to further investigate the role of Cyp2b in the development and progression of NASH because of the observed changes in serum markers of liver injury in the CDAHFD-treated groups, especially within the relatively resistant Cyp2b-null female mice. A CDAHFD caused numerous changes in gene expression relative to a ND (**S1 File**). Analysis of the differentially expressed genes between CDAHFD-fed Cyp2b-null and WT groups (log_2_FC > 1.0; **S2 File**) by hierarchial clustering revealed numerous CDAHFD-induced changes in gene expression in both female and male WT mice (**Fig 4**). Several genes that were altered by a CDAHFD in WT mice were regulated in the opposite direction in CDAHFD-fed Cyp2b-null mice (**Fig 4A and 4B**). Associated immune system genes, *Cd8* antigen beta chain 1 (*Cd8b1*) and nuclear factor of activated T-cells cytoplasmic 2 (*Nfatc2*), as well as the upstream cortisol synthesis calmodulin-like protein 4 (*Calml4*) gene had very low expression or down-regulation (blue) in CDAHFD-fed WT females but up-regulation (yellow) in Cyp2b-null counterparts (**Fig 4A**). Oppositely regulated genes between CDAHFD-fed WT and Cyp2b-null male mice included feeding behavior regulation and insulin signaling genes, hypocretin receptor 2 (*Hcrtr2*) and protein phosphatase 1 regulatory subunit 3C (*Ppp1r3c*), respectively. These genes had low expression or up-regulation (yellow) in CDAHFD-fed WT males but down-regulation (blue) in Cyp2b-null counterparts (**Fig 4B**).

Gene ontology (GO) enrichment analysis (**S3 File**) demonstrates significant (p < 0.05) increases in terms associated with lipid metabolism and immune system regulation in CDAHFD-fed Cyp2b-null female mice, and increases in stimulus/stress response, cell communication, and signal transduction terms in CDAHFD-fed Cyp2b-null male mice compared to CDAHFD-fed WT mice (**Fig 4C and 4D**). Both female and male CDAHFD-fed Cyp2b-null mice had down-regulated GO terms related to xenobiotic metabolism (**S4 Fig**). Fatty liver and NASH are known to mediate the down-regulation of detoxification enzymes, especially CYPs [51, 52]. *Cyp2b9*, *Cyp2b10*, and *Cyp2b13* were all significantly down-regulated in CDAHFD-fed WT females; conversely, *Cyp2b9* was significantly up-regulated in WT CDAHFD-fed males compared to their ND-fed counterparts (**S4 File**). Immunoblotting confirmed that CYP2B protein was reduced in female but increased in male CDAHFD-fed WT mice compared to WT ND-fed mice (**S5 Fig**). Overall, there is substantial sexual dimorphism in gene expression with Cyp2b-null females showing changes in lipid metabolism, energy metabolism, and inflammation; Cyp2b-null males showing changes related to stress responses and cell signaling (**Fig 4**).

### Analysis of fibrosis, inflammation, and stress associated biomarkers in CDAHFD-fed Cyp2b-null mice

**Fig 5:**
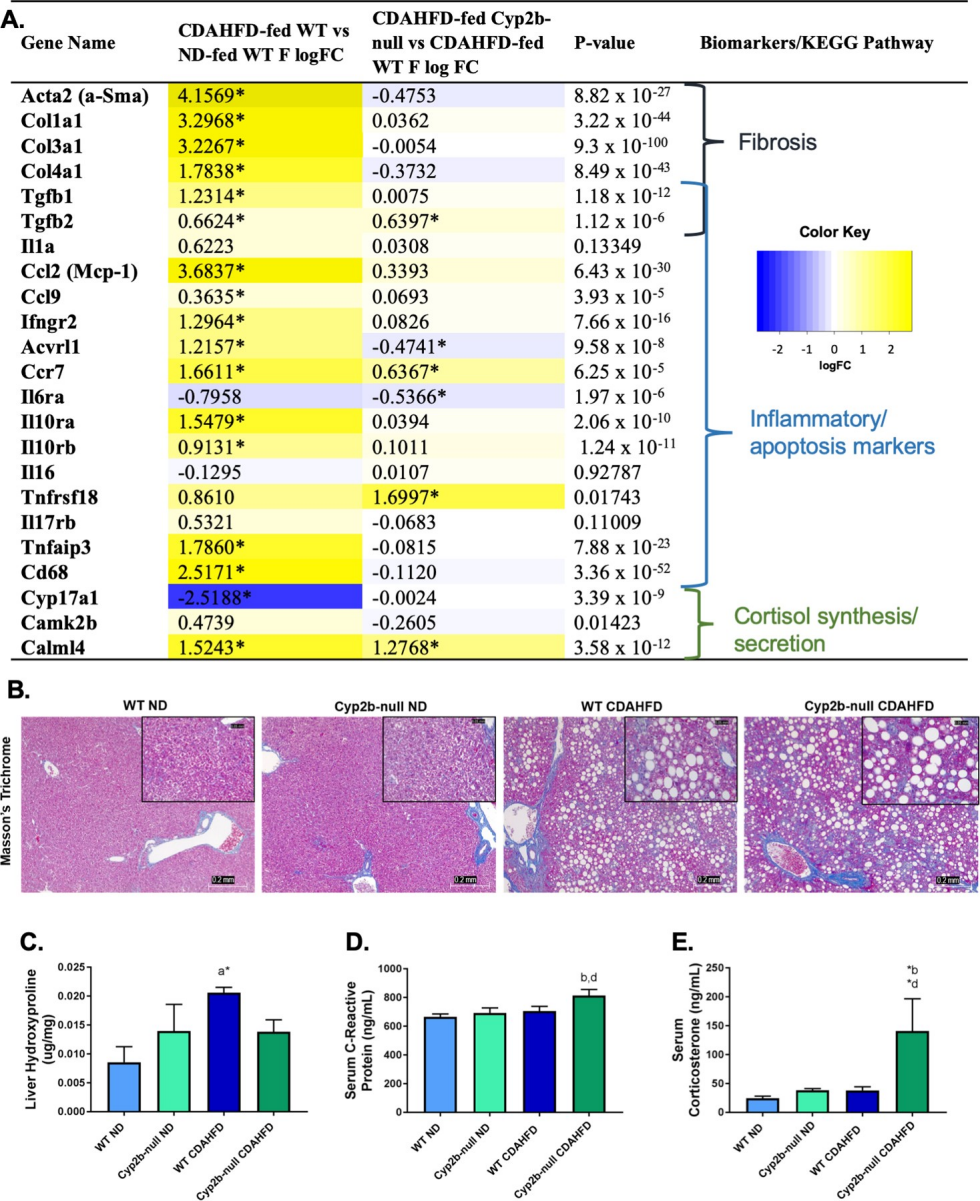
Measured liver fibrosis and inflammatory markers in CDAHFD-treated Cyp2b-null female mice. Changes in the expression of fibrosis, inflammation and stress response-associated genes were investigated and grouped by respective biomarkers and/or KEGG pathways (A). LogFC values with an asterisk indicates a significant difference (p < 0.05) between two groups, e.g. CDAHFD-fed versus ND-fed WT mice, and no asterisk denotes significance by one-way ANOVA across all treatment groups. Histopathological changes were evaluated by Masson’s trichrome staining of female liver tissues (B). Images were taken at 100x (0.2 mm) and 400x (0.05 mm) magnification. Liver hydroxyproline (C), as well as serum C-reactive protein (D) and corticosterone (E) were measured in all treatment groups. Graph data are presented as mean ± SEM. Statistical significance was determined by one-way ANOVA followed by Fisher’s LSD as the post-hoc test (n = 5). An ‘a’ indicates ND-fed WT different than CDAHFD-fed WT, ‘b’ indicates ND-fed Cyp2b-null different than CDAHFD-fed Cyp2b-null, ‘c’ indicates ND-fed WT different than ND-fed Cyp2b-null, ‘d’ indicates CDAHFD-fed WT different than CDAHFD-fed Cyp2b-null. No asterisk indicates a p-value < 0.05, * indicates a p-value < 0.01, and ** indicates a p-value < 0.0001.

Gene markers associated with fibrosis and inflammation were up-regulated in CDAHFD-fed WT female mice (**Fig 5A and S4 File**). When comparing CDAHFD-fed Cyp2b-null mice to CDAHFD-fed WT mice, expression of several fibrosis-related genes were slightly down-regulated or unchanged (**S2 File**). However, the pro-inflammatory interleukin-6 receptor (*Il6ra*) was significantly down-regulated, while other apoptotic and immune response genes such as immunosuppressive transforming growth factor beta 2 (*Tgfb2*) [53] and chemokine receptor 7 (*Ccr7*) were up-regulated in CDAHFD-fed Cyp2b-null female mice (**S5 File**). Furthermore, the tumor necrosis factor receptor superfamily member 18 (*Tnfrsf18*) also known as the glucocorticoid-induced TNFR-related protein and calmodulin-like 4 (Calml4), a calcium-binding messenger protein involved in upstream signaling of steroid hormone biosynthesis, were significantly up-regulated (logFC = 1.70 and logFC = 1.28, respectively) in CDAHFD-fed Cyp2b-null females compared to WT counterparts (**Fig 5A**), suggesting a potential role for glucocorticoid mediated suppression of inflammation and immune response.

Liver histopathology along with markers of NASH were examined because of gene expression profile differences found between CDAHFD-fed WT and Cyp2b-null mice. Masson’s Trichrome confirmed the development of cytoplasmic vacuolization and revealed the progresion of fibrosis from CDAHFD treatment; however, the difference in severity was not significant between genotypes (**Fig 5B**). Liver hydroxyproline, a major component of collagen and marker for fibrosis development, was significantly increased in female CDAHFD-fed WT mice compared to ND-fed WT mice. Hydroxyproline concentrations were 33% lower in female CDAHFD-fed Cyp2b-null mice than CDAHFD-fed WT mice; however, this was not statistically significant (p = 0.18) (**Fig 5C**). Overall, liver fibrosis was induced by a CDAHFD and some expression markers of liver fibrosis and inflammation were higher in female CDAHFD-fed WT mice than CDAHFD-fed Cyp2b-null mice; however, perturbation was often either minimal to moderate or not significant.

Liver inflammation was determined by serum levels of C-reactive protein (CRP). CDAHFD-fed Cyp2b-null females had higher (15%) CRP concentrations over WT counterparts (**Fig 5D**). Corticosterone, the main glucocorticoid in rodents, was also measured due to induction of immune system regulation-associated GO terms and inflammatory markers in CDAHFD-fed Cyp2b-null females (**Figs 4C and 5A**). Corticosterone increased in CDAHFD-fed Cyp2b-null females by 2.7X compared to WT mice (**Fig 5E**). These results concur with the up-regulation of glucocorticoid-mediated KEGG pathways including *Tnfrsf18* and *Calml4* in CDAHFD-fed Cyp2b-null females. Overall, gene expression, inflammatory markers, and corticosterone levels indicate Cyp2b-null mice differed in their response to a CDAHFD than WT mice; however, there were no significant changes in fibrosis (**Fig 5**).

**Fig 6:**
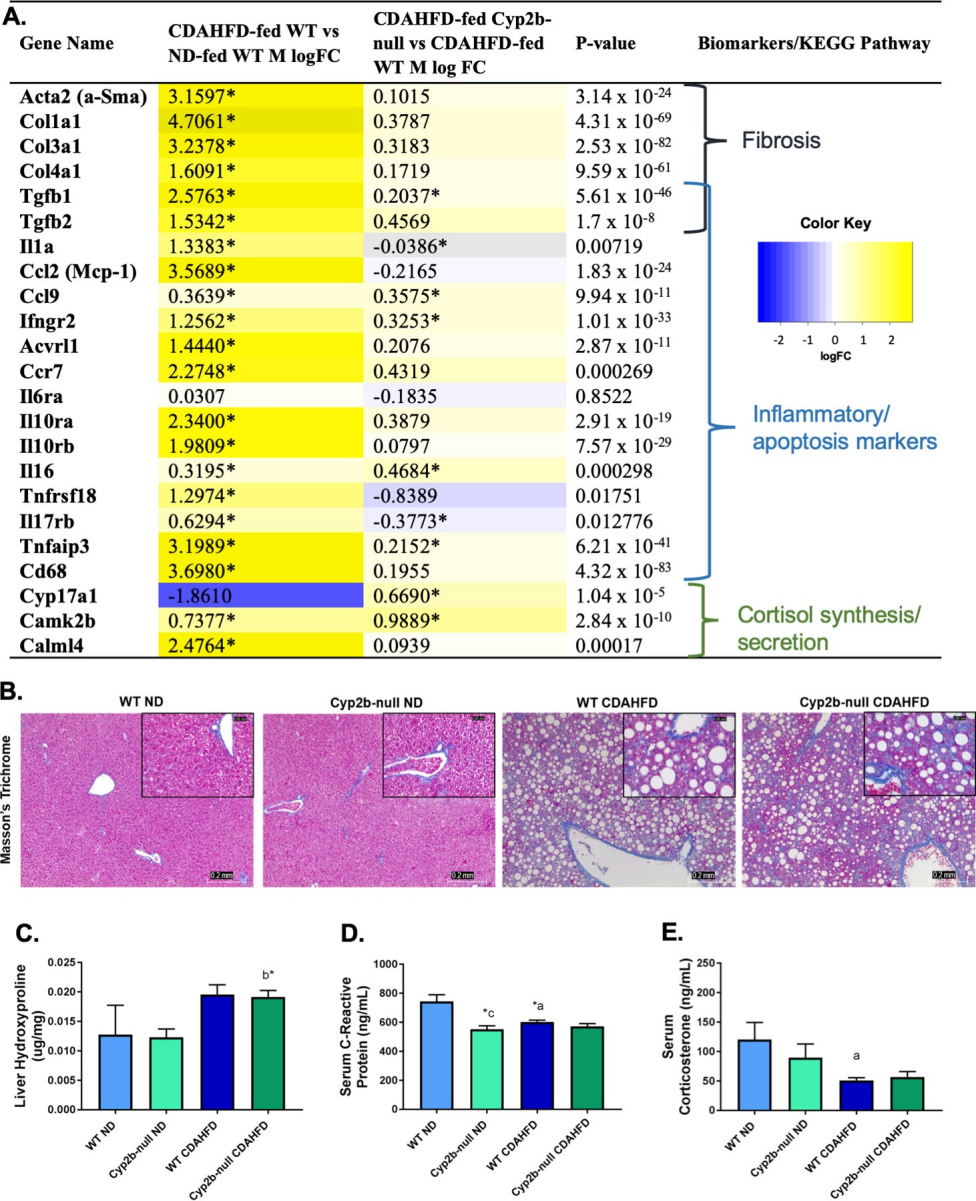
Measured liver fibrosis and inflammatory markers in CDAHFD-treated Cyp2b-null male mice. Changes in the expression of fibrosis, inflammation and stress response-associated genes were investigated and grouped by respective biomarkers and/or KEGG pathways (A). LogFC values with an asterisk indicates a significant difference (p < 0.05) between two groups, e.g. CDAHFD-fed versus ND-fed WT mice, and no asterisk denotes significance by one-way ANOVA across all treatment groups. Histopathological changes were evaluated by H&E, and Masson’s trichrome staining of male liver tissues (B). Images were taken at 100x (0.2 mm) and 400x (0.05 mm) magnification. Liver hydroxyproline (C), as well as serum C-reactive protein (D) and corticosterone (E) were measured in all treatment groups. Graph data are presented as mean ± SEM. Statistical significance was determined by one-way ANOVA followed by Fisher’s LSD as the post-hoc test (n = 5). An ‘a’ indicates ND-fed WT different than CDAHFD-fed WT, ‘b’ indicates ND-fed Cyp2b-null different than CDAHFD-fed Cyp2b-null, ‘c’ indicates ND-fed WT different than ND-fed Cyp2b-null, ‘d’ indicates CDAHFD-fed WT different than CDAHFD-fed Cyp2b-null. No asterisk indicates a p-value < 0.05, * indicates a p-value < 0.01, and ** indicates a p-value < 0.0001.

CDAHFD-fed male mice show significant adverse changes compared to ND-fed mice, with expression markers associated with increased fibrosis and inflammation up-regulated in CDAHFD-fed WT male mice and greater up-regulation of fibrosis marker genes in CDAHFD-fed Cyp2b-null males, (**Fig 6A and S2, S4, and S5 Files**) especially when compared to females (**Fig 5A**). Masson’s Trichrome staining also confirmed the development of cytoplasmic vacuolization and revealed the progression of fibrosis from CDAHFD treatment regardless of genotype (**Fig 6B and 6C**). Contrary to females, CDAHFD-fed male mice had unexpectedly lower concentrations of CRP and corticosterone in CDAHFD-treated mice (**Fig 6D and 6E**). Taken together, gene expression suggests only minor differences between CDAHFD-fed WT and Cyp2b-null males, and these changes did not manifest themselves in the histopatholgy or hydroxyproline results, indicating few to no differences between WT and Cyp2b-null male mice regarding susceptibility to NASH.

### Cyp2b-null mice show gender differences in CDAHFD-induced liver triglyceride accumulation

**Fig 7:**
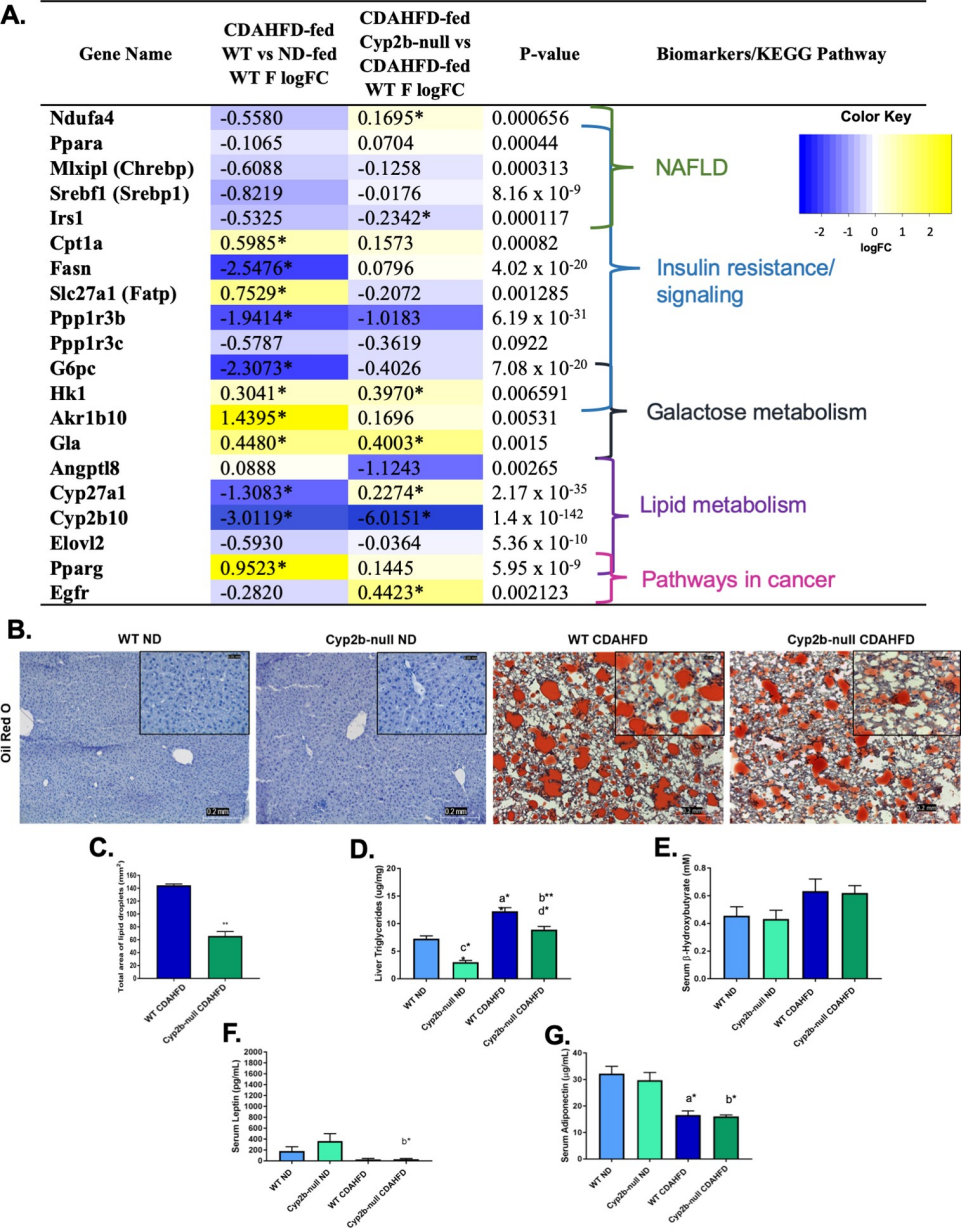
Steatosis and markers of steatosis in CDAHFD-fed WT and CDAHFD-fed Cyp2b-null female mice. Changes in the expression of nonalcoholic fatty liver disease-related genes were investigated and grouped by respective biomarkers and/or KEGG pathways (A). LogFC values with an asterisk indicates a significant difference (p < 0.05) between two groups, e.g. CDAHFD-fed versus ND-fed WT mice, and no asterisk denotes significance by one-way ANOVA across all treatment groups. Fatty liver histopathological changes were evaluated by Oil red O staining in female mice (B). Images were taken at 100x (0.2 mm) and 400x (0.05 mm) magnification. Total liver triglycerides (C) were measured in female mice to confirm Oil Red O staining results. Liver lipid droplets were also quantified by total area (D) using ImageJ Fiji from Oil Red O slides (400x). Serum levels of β-hydroxybutyrate (E), leptin (F), and adiponectin (G) were also determined. Graphed data are presented as mean ± SEM. Statistical significance was determined by one-way ANOVA followed by Fisher’s LSD as the post-hoc test (n = 5). An ‘a’ indicates ND-fed WT different than CDAHFD-fed WT, ‘b’ indicates ND-fed Cyp2b-null different than CDAHFD-fed Cyp2b-null, ‘c’ indicates ND-fed WT different than ND-fed Cyp2b-null, ‘d’ indicates CDAHFD-fed WT different than CDAHFD-fed Cyp2b-null. No asterisk indicates a p-value < 0.05, * indicates a p-value < 0.01, and ** indicates a p-value < 0.0001.

NAFLD is often a precursor to NASH and therefore differences in NAFLD were also investigated. GO analysis indicated an up-regulation of lipid metabolism-related terms in CDAHFD-fed Cyp2b-null females. In contrast, several genes associated with fatty acid metabolism were down-regulated in CDAHFD-fed WT female mice (**Fig 7A and S4 File**). Genes that reversed direction of regulation from down- to up-regulation in CDAHFD-fed Cyp2b-null female mice compared to CDAHFD-fed WT mice include Ndufa4-mitochondrial complex associated protein (*Ndufa4*) and *Cyp27a1*, which breaks down cholesterol to bile acids (**S2 and S5 Files**). Pathological analysis of Oil Red O staining established increased steatosis in CDAHFD-treated female mice, with less lipid accumulation in CDAHFD-fed Cyp2b-null females than CDAHFD-fed WT females (**Fig 7B**). Total lipid area quantified by ImageJ Fiji confirmed the pathological findings [40] that CDAHFD-fed Cyp2b-null female mice had significantly less hepatic lipids than CDAHFD-fed WT mice (**Fig 7C**) and this was verified by total triglyceride concentrations measured colorimetrically (**Fig 7D**). Despite smaller droplets (data not shown), CDAHFD-fed Cyp2b-null mice did not appear to present with microsteatosis. To make sure, serum β-hydroxybutyrate levels were also measured because impaired mitochondrial β-oxidation can cause microvesicular steatosis development [54]. Serum β-hydroxybutyrate increased in both CDAHFD-fed groups with no significant difference between genotypes (**Fig 7E**).

There were changes in the serum lipid levels of female mice (**Table 1A**). This includes decreased calcium and increased serum HDL and LDH in ND-fed Cyp2b-null mice compared to ND-fed WT mice consistent with previous data [33]. CDAHFD-fed female mice had lower serum glucose and HDL compared to ND-fed counterparts. However, between CDAHFD-fed genotypes, CDAHFD-fed Cyp2b-null mice increased HDL but decreased calcium levels. LDH was consistently increased in the serum of Cyp2b-null mice regardless of diet indicating skeletal or cardiac muscular tissue damage. Liver to serum triglyceride ratios confirm reduced accumulation of triglycerides in the livers of Cyp2b-null female mice regardless of diet (**Table 1A**). The metabolic hormones leptin and adiponectin were measured due to changes in weight, liver lipids, and gene expression changes. Significant differences were associated with diet and not genotype (**Fig 7F and 7G**).

**Table 1:** Serum biomarker levels in ND and CDAHFD-treated WT and Cyp2b-null mice.

**A.**				
**Serum Panel**	**ND-fed WT F**	**ND-fed Cyp2b-null F**	**CDAHFD-fed WT F**	**CDAHFD-fed Cyp2b-null F**
Calcium (mg/dL)	9.41 ± 0.15	8.02 ± 0.20^c^**	9.60 ± 0.14	7.72 ± 0.20^d^**
Phosphorus (mg/dL)	5.68 ± 0.38	5.95 ± 0.36	6.92 ± 0.63	7.04 ± 0.35
Glucose (mg/dL)	181.23 ± 6.72	186.42 ± 4.25	155.07 ± 12.06^a^	151.73 ± 5.41^b^*
Triglycerides (mg/dL)	59.91 ± 9.23	53.34 ± 0.40	64.88 ± 4.30	57.97 ± 7.15
Cholesterol (mg/dL)	71.88 ± 4.62	119.96 ± 42.45	70.57 ± 21.29	53.13 ± 2.02
HDL (mg/dL)	46.59 ± 2.03	51.85 ± 2.28	28.26 ± 4.97^a^*	37.60 ± 0.82^b^*^d^
LDL (mg/dL)	5.6 ± 0.20	8.13 ± 2.64	6.58 ± 2.19	4.28 ± 0.98
VLDL (mg/dL)	11.98 ± 1.85	10.67 ± 1.45	12.98 ± 0.86	11.59 ± 1.43
LDH (U/L)	n.d.	260.73 ± 104.64^c^*	n.d.	680.96 ± 96.79 ^b^^d^**
Direct bilirubin (mg/dL)	0.024 ± 0.004	0.04 ± 0.01	0.16 ± 0.11	0.068 ± 0.01
Indirect bilirubin (mg/dL)	0.218 ± 0.05	0.78 ± 0.58	0.56 ± 0.24	0.21 ± 0.048
Total bilirubin (mg/dL)	0.24 ± 0.05	0.69 ± 0.47	0.71 ± 0.22	0.45 ± 0.17
Liver:Serum Triglycerides	0.139 ± 0.03	0.065 ± 0.01^c^	0.192 ± 0.02	0.163 ± 0.02^b^*
**B.**				
**Serum Panel**	**ND-fed WT M**	**ND-fed Cyp2b-null M**	**CDAHFD-fed WT M**	**CDAHFD-fed Cyp2b-null M**
Calcium (mg/dL)	9.82 ± 0.27	8.54 ± 0.17^c^*	9.26 ± 0.10	8.50 ± 0.39
Phosphorus (mg/dL)	5.69 ± 0.34	5.76 ± 0.37	6.72 ± 0.21	7.37 ± 0.51^b^*
Glucose (mg/dL)	206.87 ± 15.42	212.36 ± 7.55	124.80 ± 3.55^a^**	141.45 ± 9.21^b^*
Triglycerides (mg/dL)	96.24 ± 5.46	66.72 ± 4.59^c^*	53.004 ± 4.64^a^**	55.15 ± 3.99
Cholesterol (mg/dL)	96.18 ± 4.65	105.48 ± 3.69	38.26 ± 1.78^a^**	46.38 ± 3.078^b^**
HDL (mg/dL)	69.76 ± 3.24	77.94 ± 2.25^c^	18.86 ± 2.33^a^**	29.53 ± 2.46^b^**^d^
LDL (mg/dL)	3.53 ± 0.20	3.29 ± 0.22	6.19 ± 2.09	3.40 ± 0.56
VLDL (mg/dL)	19.25 ± 1.09	13.34 ± 0.92^c^*	10.6 ± 0.93^a^**	11.03 ± 0.80
LDH (U/L)	n.d.	187.64 ± 23.73^c^**	n.d.	909.80 ± 55.91^b^^d^**
Direct bilirubin (mg/dL)	0.02 ± 0.0045	0.026 ± 0.004	0.12 ± 0.0045^a^**	0.095 ± 0.013^b^**^d^
Indirect bilirubin (mg/dL)	0.14 ± 0.014	0.15 ± 0.0097	0.22 ± 0.015^a^*	0.3 ± 0.026^b^**^d^*
Total bilirubin (mg/dL)	0.16 ± 0.012	0.18 ± 0.0073	0.34 ± 0.017^a^**	0.41 ± 0.032^b^**^d^
Liver:Serum Triglycerides	0.081 ± 0.009	0.115 ± 0.007	0.244 ± 0.012^a^**	0.322 ± 0.034^b^**^d^

Data are presented as mean ± SEM. Statistical significance was determined by one-way ANOVA followed by Fisher’s LSD as the post-hoc test (n = 5).

‘a’ indicates ND-fed WT different than CDAHFD-fed WT.

‘b’ indicates ND-fed Cyp2b-null different than CDAHFD-fed Cyp2b-null.

‘c’ indicates ND-fed WT different than ND-fed Cyp2b-null.

‘d’ indicates CDAHFD-fed WT different than CDAHFD-fed Cyp2b-null.

No asterisk next to a ‘letter’ indicates a p-value < 0.05.

* indicates a p-value < 0.01, and.

** indicates a p-value < 0.0001. n.d. = not detected.

Raw data from the serum panel and other measured endpoints are supplied in **S6 File**.

**Fig 8:**
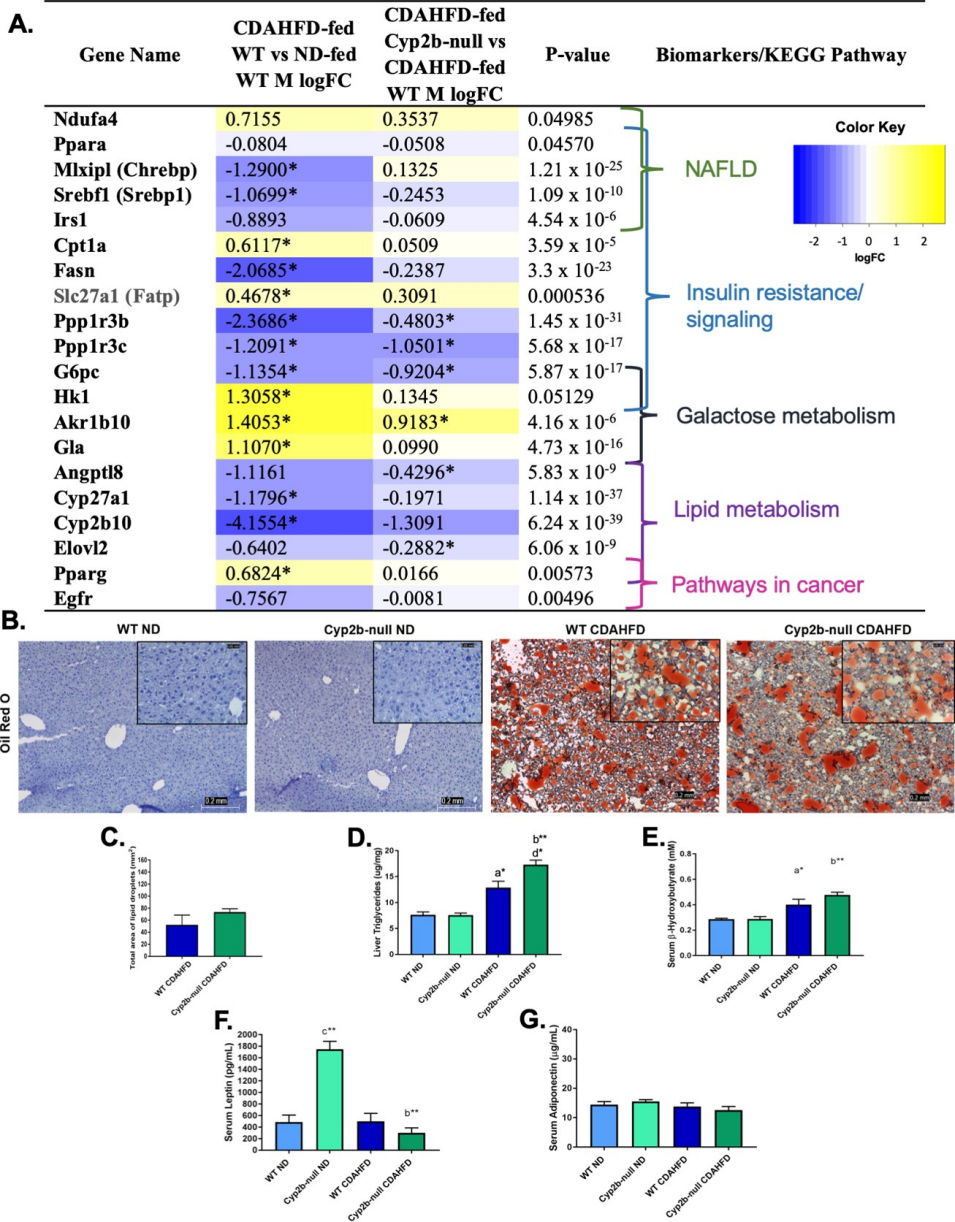
Steatosis and markers of steatosis in CDAHFD-fed WT and CDAHFD-fed Cyp2b-null male mice. Changes in the expression of nonalcoholic fatty liver disease-related genes were investigated and grouped by respective biomarkers and/or KEGG pathways (A). LogFC values with an asterisk indicates a significant difference (p < 0.05) between two groups, e.g. CDAHFD-fed versus ND-fed WT mice, and no asterisk denotes significance by one-way ANOVA across all treatment groups. Fatty liver histopathological changes were evaluated by Oil red O staining in female mice (B). Images were taken at 100x (0.2 mm) and 400x (0.05 mm) magnification. Total liver triglycerides (C) were measured in male mice to confirm Oil Red O staining results. Liver lipid droplets were also quantified by total area (D) using ImageJ Fiji from Oil Red O slides (400x). Serum levels of β-hydroxybutyrate (E), leptin (F), and adiponectin (G) were also determined. Graph data are presented as mean ± SEM. Statistical significance was determined by one-way ANOVA followed by Fisher’s LSD as the post-hoc test (n = 5). An ‘a’ indicates ND-fed WT different than CDAHFD-fed WT, ‘b’ indicates ND-fed Cyp2b-null different than CDAHFD-fed Cyp2b-null, ‘c’ indicates ND-fed WT different than ND-fed Cyp2b-null, ‘d’ indicates CDAHFD-fed WT different than CDAHFD-fed Cyp2b-null. No asterisk indicates a p-value < 0.05, * indicates a p-value < 0.01, and ** indicates a p-value < 0.0001.

Numerous genes associated with fatty acid metabolism were also down-regulated in CDAHFD-fed WT male mice compared to ND-fed counterparts, and these genes were further down-regulated in CDAHFD-fed Cyp2b-null male mice (**Fig 8A and S2, S4, and S5 Files**), suggesting greater steatosis in the CDAHFD-fed Cyp2b-null male mice. These genes include regulators of glycogen metabolism, Ppp1r3b and Ppp1r3c, glucose metabolism regulator, glucose-6-phosphatase (*G6pc*), serum triglyceride regulator, angiopoietin-like protein 8 (*Angptl8*), and long chain fatty acid elongase 2 (*Elovl2*). Liver steatosis also increased in CDAHFD-fed male mice, with higher liver triglyceride levels in Cyp2b-null compared to WT mice (**Fig 8B–8D**) corroborating the gene expression data. Serum β-hydroxybutyrate also indicates no change in ketosis in male mice similar to female mice (**Fig 8E**).

ND-fed Cyp2b-null male mice showed significantly lower serum calcium and triglycerides consistent with previous data [33], and greater HDL and LDH similar to females. Relative to CDAHFD-fed WT mice, CDAHFD-fed Cyp2b-null mice showed greater HDL, bilirubin, serum: triglyceride ratios, and LDH. Males showed similar directional changes in HDL and LDH as females; however, their serum: triglyceride ratios were in opposing directions, which was expected because liver triglycerides went down in CDAHFD-fed Cyp2b-null females and up in CDAHFD-fed Cyp2b-null males (**Table 1B**). Serum leptin increased 2.6-fold in ND-fed Cyp2b-null males compared to their WT counterparts, while leptin levels remained low in CDAHFD-fed mice (**Fig 8F**). No differences were observed between groups for male adiponectin levels (**Fig 8G**). Overall, Cyp2b-null female and male mice reacted very differently to a CDAHFD pertaining to liver lipids, with protection from steatosis in Cyp2b-null females and increased steatosis in Cyp2b-null males.

### qPCR confirmation of changes in gene expression

**Fig 9:**
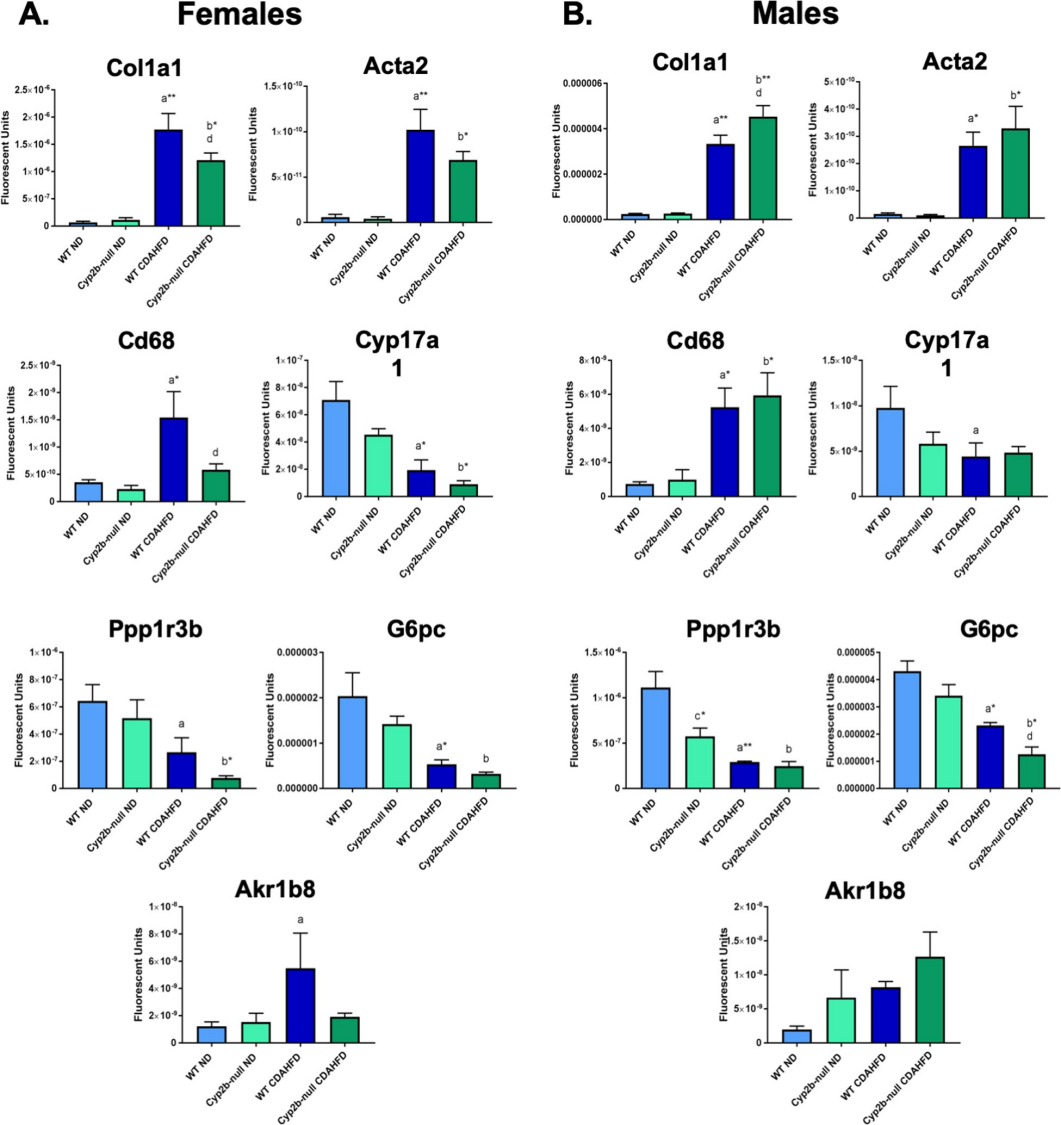
qPCR confirmation of RNAseq analysis. Changes in the expression of genes in females (A) and males (B) involved in fibrosis, inflammation, insulin signaling, fatty liver, and proliferation by qPCR confirmation. Data are presented as mean ± SEM. Statistical significance was determined by one-way ANOVA followed by Fisher’s LSD as the post-hoc test (n = 5). An ‘a’ indicates ND-fed WT different than CDAHFD-fed WT, ‘b’ indicates ND-fed Cyp2b-null different than CDAHFD-fed Cyp2b-null, ‘c’ indicates ND-fed WT different than ND-fed Cyp2b-null, ‘d’ indicates CDAHFD-fed WT different than CDAHFD-fed Cyp2b-null. No asterisk indicates a p-value < 0.05, * indicates a p-value < 0.01, and ** indicates a p-value < 0.0001.

qPCR was used to verify RNAseq results of genes associated with hepatic fibrosis (*Acta2; Col1a1*), inflammation (*Cd68*) and steroid metabolism associated with cortisol (*Cyp17a1*), insulin signaling and glucose metabolism (*Ppp1r3b*; *G6pc*), and cell proliferation (aldo-keto reductase 1b8, *Akr1b8*, ortholog of *AKR1B10*) in female (**Fig 9A**) and male (**Fig 9B**) mice. All genes verified by qPCR exhibited similar directional trends in terms of gene expression to the RNAseq results (**Figs 5–8 and S1 File**). *Col1a1* was significantly different between CDAHFD-fed WT and CDAHFD-fed Cyp2b-null mice in both sexes, with lower *Col1a1* expression in the Cyp2b-null females (**Fig 9A**) and higher in the Cyp2b-null males provided a CDAHFD (**Fig 9B**). *Acta2* was not different between CDAHFD-treated genotypes but showed similar trends in expression to *Col1a1*, suggesting increased fibrosis. *Cd68* also differed by genotype in CDAHFD-fed female mice only, with decreased expression in CDAHFD-fed Cyp2b-null mice, suggesting lower inflammation and macrophage infiltration in these mice. *Cyp17a1* qPCR results were similar to RNAseq, however, there was no statistical differences between CDAHFD-fed WT and Cyp2b-null mice. The insulin signaling and glucose metabolism genes, *Ppp1r3b* and *G6pc* trended in the same direction of the RNAseq results, with decreased G6pc expression in both CDAHFD-fed Cyp2b-null female and male mice compared to WT counterparts; however only males were significant by ANOVA. Females were significant by t-tests directly comparing CDAHFD-fed WT to CDAHFD-fed Cyp2b-null mice (p = 0.04). *Akr1b8* showed similar patterns in gene expression to RNAseq, but no differences between genotypes in CDAHFD-fed mice. In conclusion, qPCR results showed exactly the same trends as RNAseq but did not always agree statistically. qPCR also indicates an increase in fibrosis, inflammation, and fatty liver in CDAHFD-fed mice that is repressed somewhat in Cyp2b-null females.

## Discussion

Cyp2b-null female mice weigh less, primarily due to lower white adipose tissue mass, and are protected from diet-induced steatosis and to a lesser extent diet-induced NASH. There were few differences in fibrosis markers between CDAHFD-fed WT and Cyp2b-null females, however hydroxyproline levels were slighty lower in Cyp2b-null female mice. CDAHFD caused substantial liver injury, however Cyp2b-null females had significantly lower markers of liver injury including ALT, AST, and ALP compared to their WT counterparts. Protection from liver damage in Cyp2b-null females may occur due to lower immune suppression as indicated by gene expression changes in Tgfb2, Il6ra, and activin receptor-like kinase 1 (Acvrl1), potentially due to glucocorticoid-mediated repression.

In contrast, CDAHFD-fed Cyp2b-null male mice exhibit no significant changes in serum ALT, AST, and ALP in comparison to CDAHFD-fed WT mice indicating no differences in liver damage. Fibrosis was heavily induced by a CDAHFD, but there was also no difference between CDAHFD-fed male groups except for PCNA. Cyp2b-null males also have slightly greater inflammatory responses based on RNA-seq data with no changes in CRP or corticosterone levels. CDAHFD-fed Cyp2b-null males also showed a 1.5-fold increase of serum creatine kinase levels compared to all other treatment groups, suggesting that CDAHFD treatment causes damage to Cyp2b-null males in other tissues, such as cardiac or skeletal muscle, in addition to liver [50].

Direct measurement of liver triglycerides and histopathological staining using Oil Red O revealed less lipid accumulation in the livers of CDAHFD-fed Cyp2b-null females than CDAHFD-fed WT females. Liver to serum triglyceride ratios also indicate less triglyceride accumulation in the livers of Cyp2b-null female mice (especially when treated with CDAHFD) compared to their WT counterparts. GO enrichment analysis of RNA-seq data demonstrated significant increases in terms associated with lipid metabolism in CDAHFD-fed Cyp2b-null female mice compared to CDAHFD-fed WT female mice. Several genes involved in fatty acid metabolism were down-regulated in CDAHFD-fed WT females. When comparing gene expression of CDAHFD-fed Cyp2b-null mice to their WT counterparts, lipid metabolism genes were either minimally down-regulated but not significantly, or reversed direction and were slightly up-regulated as shown in Fig 7.

Conversely, male Cyp2b-null mice were not protected from development of fatty liver. Consistent with previous results [33], liver steatosis increased in CDAHFD-fed male mice, with more triglyceride accumulation in CDAHFD-fed Cyp2b-null males than CDAHFD-fed WT males as demonstrated by liver triglycerides and liver to serum triglyceride ratios. CDAHFD-fed Cyp2b-null male mice also had more down-regulated lipid metabolism associated genes compared to female counterparts, with several genes further down-regulated from CDAHFD-fed WT males, including regulators of glycogen and triglyceride metabolism. Based on these results, it is clear that a lack of hepatic Cyp2b members is not protective against fatty liver disease in male mice.

The progression of NAFLD to NASH is characterized by inflammation, fibrosis, stress responses, and hepatocellular injury [5]. GO terms related to xenobiotic metabolism were down-regulated in female and male CDAHFD-fed Cyp2b-null mice in comparison to CDAHFD-fed WT mice, which is commonly seen in NASH [51, 52]. Interestingly, CDAHFD decreased Cyp2b expression in WT females, but increased Cyp2b expression in WT males. This sexual dimorphism of Cyp2b gene expression in WT mice fed a CDAHFD may be associated with the gender differences observed in Cyp2b-null mice for other measured biomarkers. Markers of fibrosis and inflammation were also examined in CDAHFD-fed mice, however changes in these markers were relatively small. Histopathology revealed development of cytoplasmic vacuolization and fibrosis from CDAHFD treatment in both sexes, but no significant differences between genotypes. Of the major fibrotic markers investigated, only *Tgfb1* and *Tgfb2* were perturbed in CDAHFD-fed Cyp2b-null mice compared to CDAHFD-fed WT mice; *Tgfb1* in males and *Tgfb2* in females. Consequently, susceptibility to NASH only differed following the CDAHFD, but not by genotype despite minor respression of inflammatory markers and hydroxyproline in female mice.

The hepatic Cyp2b members, *Cyp2b9* and *Cyp2b13* are highly sexually dimorphic [55–57], and CAR regulation of several genes including *Cyp2b10* is also sexually dimorphic as is cell proliferation [58, 59]. Signal transducer and activator of transcription 5b (Stat5b) mediates growth hormone (GH)-dependent sexually dimorphic gene expression in the male liver [60], and results indicate that the GH-STAT5b pathway regulates female predominant expression of *Cyp2b9*, as this pathway is suppressed by the male GH secretion profile [61, 62] through its regulation of HNF4α, CAR, and forkhead box A2 (FoxA2) [55, 57, 63]. Female predominant regulation of these genes is crucial in their physiological responses. For example, FoxA2 increases fatty acid oxidation, decreases obesity, regulates *Cyp2b9*, and represses hepatocellular carcinoma only in female mice [63–65]. However, it’s ability to regulate obesity in a sexually dimorphic manner has not been established [66]. Based on this information, it is certainly possible that the Cyp2b’s play a role in lipid metabolism that decreases obesity, provides protection from liver toxicity, but increases NAFLD in female mice because of their much higher expression, greater control, and production of key but as yet not completely understood oxylipins [21]. Most NAFLD studies using mouse models have found that disease is more severe in males, however sex differences differ by model and strain [67]. For example, a recent study found female C57Bl/6J mice fed a high-fructose diet to be more susceptible to NAFLD in terms of greater hepatic inflammation and decreased adiponectin in visceral adipose tisse than males, with no difference in liver steatosis between genders [68].

These gender differences are also seen in human CYP2B6, as it is also predominantly expressed in females although to a much lesser extent [69]. Current studies investigating the effects of NAFLD and NASH on CYP2B6 expression, activity and protein levels are inconclusive. A slight increase in the mRNA levels of CYP2B6 was found in steatotic and NASH human liver tissues, with no change in protein level or activity [70]. Conversely, the progression of NAFLD to hepatocellular carcinoma drastically decreased the estimated activity of CYP2B6 in hepatocellular carcinoma patients [71]. To our knowledge, CYP2B6 expression in NAFLD and NASH patients based on gender has yet to be determined; however, it is established that premenopausal women are protected from dysmetabolism, and NAFLD more often affects men [72].

A previous diet-induced obesity study in our laboratory performed with a 60% HFD for 10-weeks increased obesity in Cyp2b-null males only [33]; however, WAT was increased in both genders. Liver weight was decreased in Cyp2b-null males regardless of diet (ND or HFD), while liver: serum triglyceride ratios increased in Cyp2b-null males. Furthermore, serum cholesterol, which is associated with progressive NAFLD and potentially NASH [73, 74] was increased in males, but unaffected in females [33]. Taken together, Cyp2b-null males were susceptible to obesity and NAFLD with markers indicating the potential for progressive liver disease with the exception of inflammation. Females showed significantly fewer effects including those on the liver. It is because of this lack of inflammatory markers plus increased liver triglycerides providing protection from free fatty acid-induced oxidative stress observed in the previous study, that we hypothesized less progression to NASH. Interestingly, our hypothesis is correct although the manner of progression was not expected. In Cyp2b-null females, we observed protection from a progressive increase in NASH biomarkers, but that was associated with a decrease in NAFLD following the CDAHFD. In Cyp2b-null males, very few differences in NASH markers were observed while NAFLD increased; identical to the previous study. This suggests that the lack of Cyp2b may increase NAFLD, especially in males, but is protective from progression of the disease to NASH although the data with males is equivocal and in females is may be due to protection from initial damage and NAFLD.

In conclusion, the data presented indicates CDAHFD-fed Cyp2b-null female mice are less susceptible to the development of obesity and NAFLD than WT mice, and have less inflammation potentially due to glucocorticoid-mediated repression of immune responses. Female Cyp2b-null mice fed CDAHFD had decreased concentrations of serum liver injury biomarkers (ALT, AST, and ALP) and less liver steatosis compared to their WT CDAHFD-fed counterparts. In contrast, male Cyp2b-null mice are more susceptible to liver damage and hepatic steatosis with no changes in fibrosis or inflammation markers between CDAHFD-fed WT and Cyp2b-null mice. CDAHFD-fed Cyp2b-null male mice had more liver triglycerides accumulation, as well as significantly higher concentrations of circulating creatine kinase compared to CDAHFD-fed WT males, indicating damage in other tissues besides the liver. Taken together, there are marked gender-based differences in the role of Cyp2b in the development of NAFLD and progression to NASH.

## Supporting information

S1 Table Primers and annealing temperatures for qPCR. (PDF)

S1 Fig Timeline of procedures performed. Procedures performed during an 8-week treatment of 9–10 week old WT and Cyp2b-null mice with either a normal diet (ND; 6.2% fat) or a choline-deficient, L-amino acid-defined high fat diet (CDAHFD; 62% fat and 0.1% methionine).
(JPEG)

S2 Fig Feed consumption of WT and Cyp2b-null mice during 8-weeks of diet-induced NASH treatment. Female (A) and male (B) feed consumption was measured by weighing the food every alternate day. Data are presented as mean calories ± SEM. Statistical significance was determined by one-way ANOVA followed by Fisher’s LSD as post-hoc test (n = 9 An ‘a’ indicates ND-fed WT different than CDAHFD-fed WT, ‘b’ indicates ND-fed Cyp2b-null different than CDAHFD-fed Cyp2b-null, ‘c’ indicates ND-fed WT different than ND-fed Cyp2b-null, ‘d’ indicates CDAHFD-fed WT different than CDAHFD-fed Cyp2b-null.
(JPEG)

S3 Fig Mice group by diet over genotype based on their gene expression profile. Heat maps showing log2-transformed, Z-score scaled RNA-Seq expression of 500 genes in female (A) and male (B) mice, with the highest variance between treatment groups. Red and blue color intensity indicate gene up- or down-regulation, respectively. Dendrogram clustering on the x-axis indicates sample similarity, whereas dendrogram clustering on the y-axis groups genes by expression profile across samples.
(JPEG)

S4 Fig Gene ontology (GO) term enrichment analysis summary for down-regulated GO terms in CDAHFD-fed Cyp2b-null mice. GO term enrichment analysis summary using Revigo [43] for significant down-regulated GO terms in CDAHFD-fed Cyp2b-null female (A) and male (B) mice compared to CDAHFD-fed WT mice. Each scatterplot contains enriched GO terms from the biological process class that remain after term redundancy is reduced and are displayed in a two-dimensional space where semantically similar GO terms are positioned closer together within the plot. Each circle represents an enriched GO term; the cooler the color of a term, the greater signficance (p < 0.05) of that term with measured changes in gene expression. Circle size indicates the frequency of the GO term in the underlying GO database, i.e. circles of more general terms are larger.
(JPEG)

S5 Fig Immunoblots of Cyp2b protein expression between ND-fed and CDAHFD-fed WT and Cyp2b-null mice. Microsomes were prepared by homogenizing frozen livers followed by differential centrifugation as described previously [75]. Protein concentrations were determined using Bradford reagent (Bio-Rad). Immunoblots were performed using 30 μg of microsomal protein separated on 12% SDS-polyacrylamide gels (BioRad). Protein was transferred onto 0.2μm polyvinylidene difluoride (PVDF) membrane and were recognized using polyclonal antibodies to Cyp2b (previously developed in house) [18, 76]. β-actin (Sigma Aldrich, St. Louis MO USA) was used as the reference protein. Chemiluminescent immunoblot detection was done using alkaline phosphatase conjugated secondary antibodies, where in anti-mouse IgG (Immunostar, Bio-Rad) was used to visualize β-actin and anti-rabbit IgG (Immunostar, Bio-Rad) was used to visualize Cyp2b. Protein was quantified by densitometry (Image Lab 6.0.1, BioRad, Hercules, CA). Relative density is shown as the average of two samples using β-actin as the reference gene. Data are presented as relative mean of WT ND compared to each treatment group. Statistical significance was determined by one-way ANOVA followed by Fisher’s LSD as post-hoc test (n = 2). An ‘a’ indicates WT ND are different than WT CDAHFD, ‘b’ indicates Cyp2b-null ND are different than Cyp2b-null CDAHFD, ‘c’ indicates Cyp2-null ND are different than WT ND, ‘d’ indicates WT CDAHFD are different than Cyp2b-null CDAHFD. No asterisk indicates a p-value < 0.05, * indicates a p-value < 0.01, and ** indicates a p-value < 0.0001.
(JPG)

S6 Fig Full immunoblot and gel images required by PLoS ONE. Full immunoblot images of PCNA, CYP2B, and β-actin. (A) CYP2B immunoblot: CYP2B is sexually dimorphic and expressed much higher in females than males [55–57]. (B) Microsomal β-actin as the housekeeping protein. (C) PCNA. (D) Nuclear β-actin as the housekeeping protein. Left hand side of blots are often but not always stained with molecular weight markers. Blot images are from **S5 Fig** (CYP2B and β-actin) and **Fig 1**(PCNA and β-actin).
(PDF)

S1 File List of differentially expressed genes by multiple comparisons for all treatment groups. Up- and down-regulated differentially expressed genes from raw read counts were determined by multiple comparisions in EdgeR for all treatment groups (p < 0.05, FDR < 0.1).
(XLSX)

S2 File Differentially expressed gene list of CDAHFD-fed Cyp2b-null mice compared to CDAHFD-fed WT mice. Normalized counts of genes from the multiple comparisions results were compared by Student’s t tests to determine significant (p < 0.05) differentially expressed genes between CDAHFD-fed Cyp2b-null and WT groups.
(XLSX)

S3 File GO term enrichment analysis list of up and down-regulated genes in CDAHFD-fed Cyp2b-null mice compared to CDAHFD-fed WT mice. GOSeq [42], a GO term enrichment analysis program was used to adjust for gene length and expression bias of significant differentially expressed genes between CDAHFD-fed groups in female and male mice.
(XLSX)

S4 File Differentially expressed gene list of CDAHFD-fed WT mice compared to ND-fed WT mice. Normalized counts of genes from the multiple comparisions results were compared by Student’s t tests to determine significant (p < 0.05) differentially expressed genes between ND-fed and CDAHFD-fed WT groups.
(XLSX)

S5 File List of altered KEGG pathways in CDAHFD-fed Cyp2b-null mice compared to CDAHFD-fed WT mice. Significant differentially expressed genes between CDAHFD-fed Cyp2b-null and WT groups were annotated to NCBI Gene IDs using InterPro and entered into KEGG Mapper [45].
(XLSX)

S6 File Raw data from necropsies, glucose tolerance tests, serum and liver biomarkers, and qPCR. (XLSX)
