# Supplementary material for: Gender differences in diet-induced steatotic disease in Cyp2b-null mice
Source: PLoS One. 2020 Mar 10;15(3):e0229896. doi: 10.1371/journal.pone.0229896 (PMC7064244; doi:10.1371/journal.pone.0229896)
Supplement: S1 Table — (PDF) [file pone.0229896.s001.pdf]

S1 Table. Primer sequences used to determine changes in gene expression by qPCR.

Gene	Forward Primer	Reverse Primer	T_a(°C)
18s	ATGGCCGTTCTTAGTTGGTG	ATGCCAGAGTCTCGTTCGTT	64
Acta2	CGAAACCACCTATAACAGCATCA	GCGTTCTGGAGGGGCAAT	57
Akr1b8	TCAGCCCACGAGGCTTCCTTC	CTCCGGAGTCGCATTTGCTCGCA	66
Cd68	CGCAGACGACAATCAACCTA	AGTGGCATGGTGAAGAGATG	59
Col1a1	GAGAGCGAGGCCTTCCCGGA	GGGAGCCAGCGGGACCTTGT	66
Cyp17a1	GATCGGTTTATGCCTGAGCG	TCCGAAGGGCAAATAACTGG	61
G6pc	CGACTCGCTATCTCCAAGTGA	GGGCGTTGTCCAAACAGAAT	56
Ppp1r3b	AGCCGTACAATGGACCAGAT	AGTAGTAGGGCCCCAGCTTT	62.4
