# Supplementary figures and images for: Gender differences in diet-induced steatotic disease in Cyp2b-null mice
Source: PLoS One. 2020 Mar 10;15(3):e0229896. doi: 10.1371/journal.pone.0229896 (PMC7064244; doi:10.1371/journal.pone.0229896)

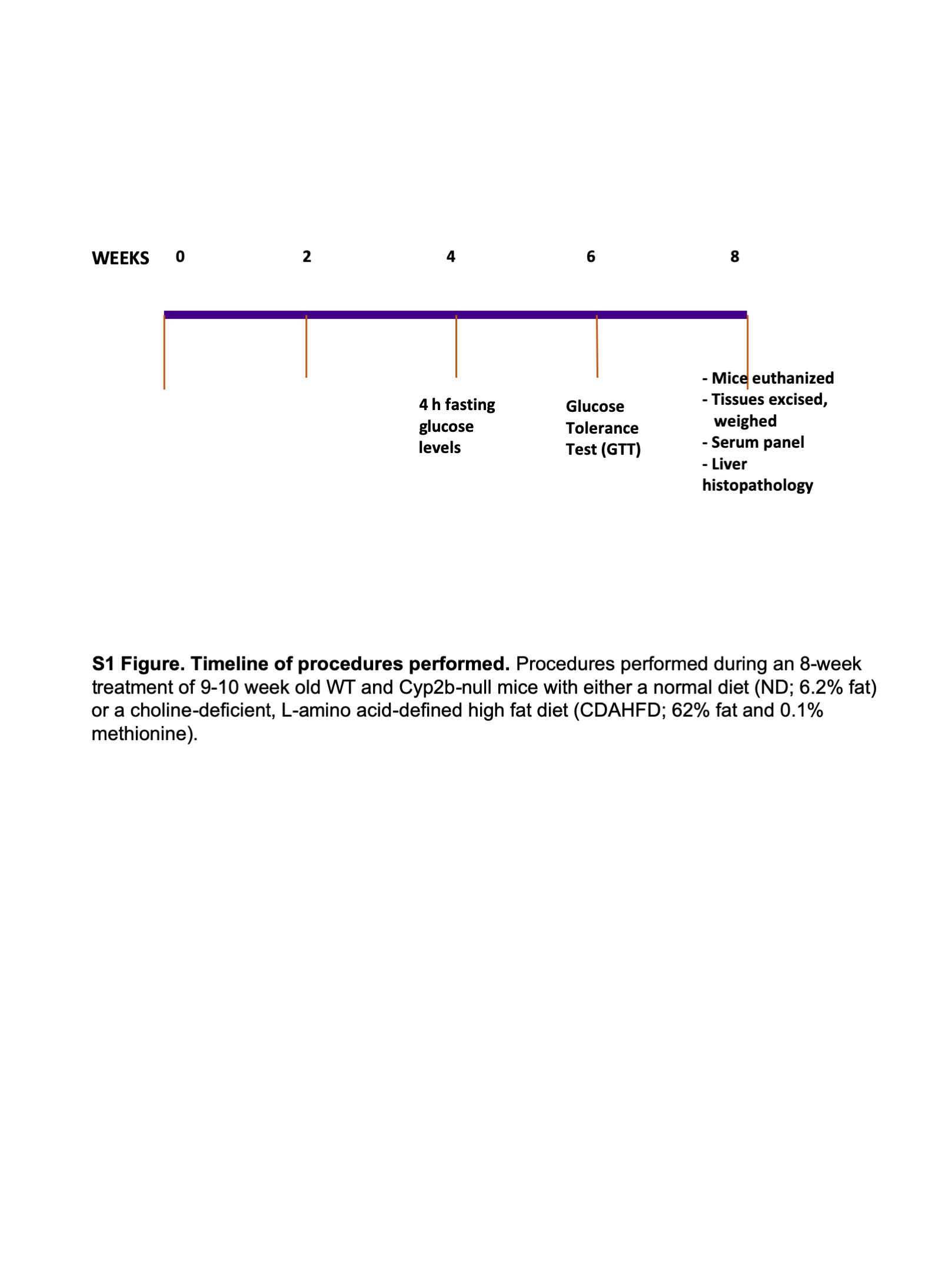

Supplement: S1 Fig — Procedures performed during an 8-week treatment of 9–10 week old WT and Cyp2b-null mice with either a normal diet (ND; 6.2% fat) or a choline-deficient, L-amino acid-defined high fat diet (CDAHFD; 62% fat and 0.1% methionine). (JPEG) [file pone.0229896.s002.jpeg]

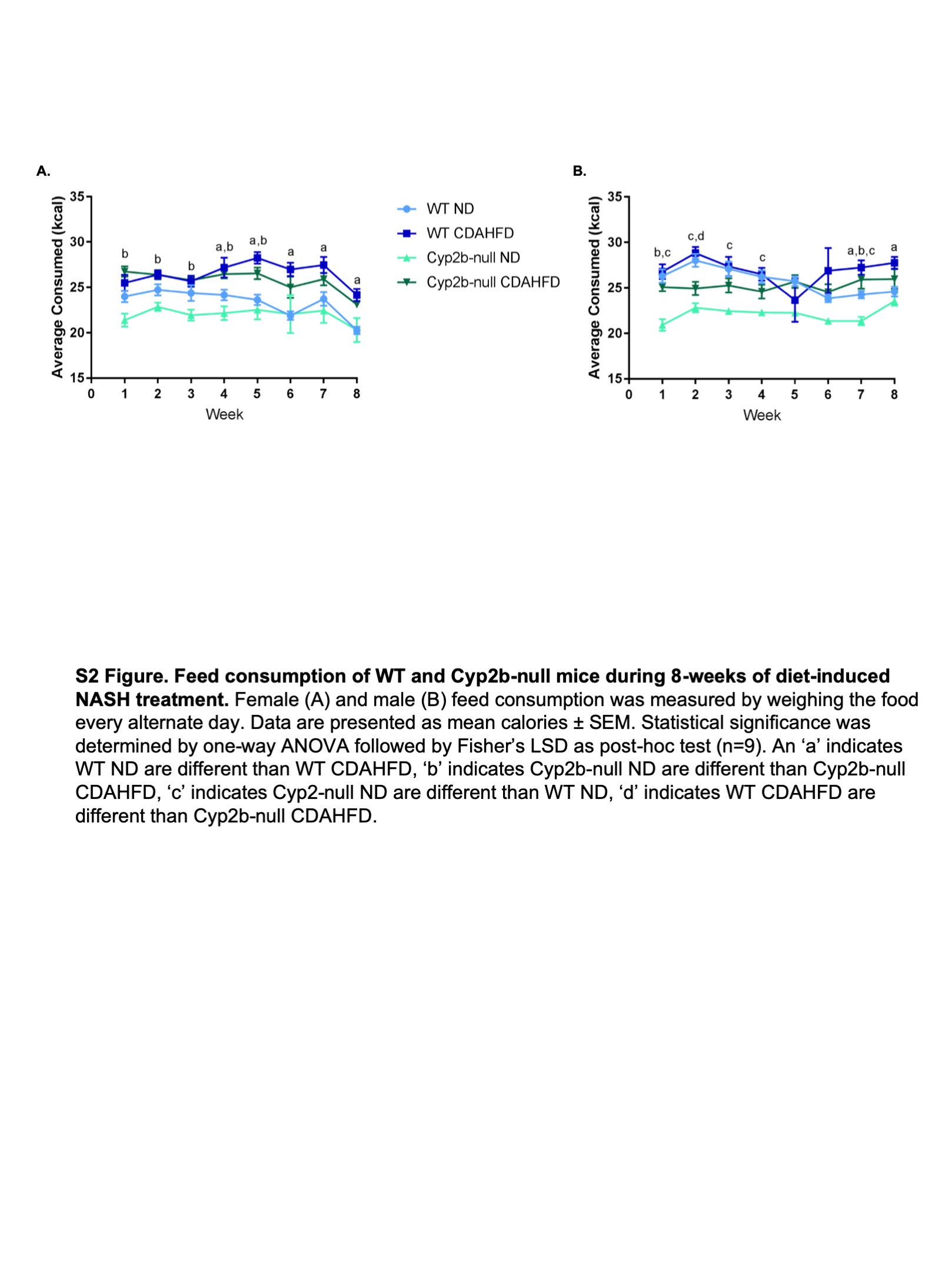

Supplement: S2 Fig — Female (A) and male (B) feed consumption was measured by weighing the food every alternate day. Data are presented as mean calories ± SEM. Statistical significance was determined by one-way ANOVA followed by Fisher’s LSD as post-hoc test (n = 9 An ‘a’ indicates ND-fed WT different than CDAHFD-fed WT, ‘b’ indicates ND-fed Cyp2b-null different than CDAHFD-fed Cyp2b-null, ‘c’ indicates ND-fed WT different than ND-fed Cyp2b-null, ‘d’ indicates CDAHFD-fed WT different than CDAHFD-fed Cyp2b-null. (JPEG) [file pone.0229896.s003.jpeg]

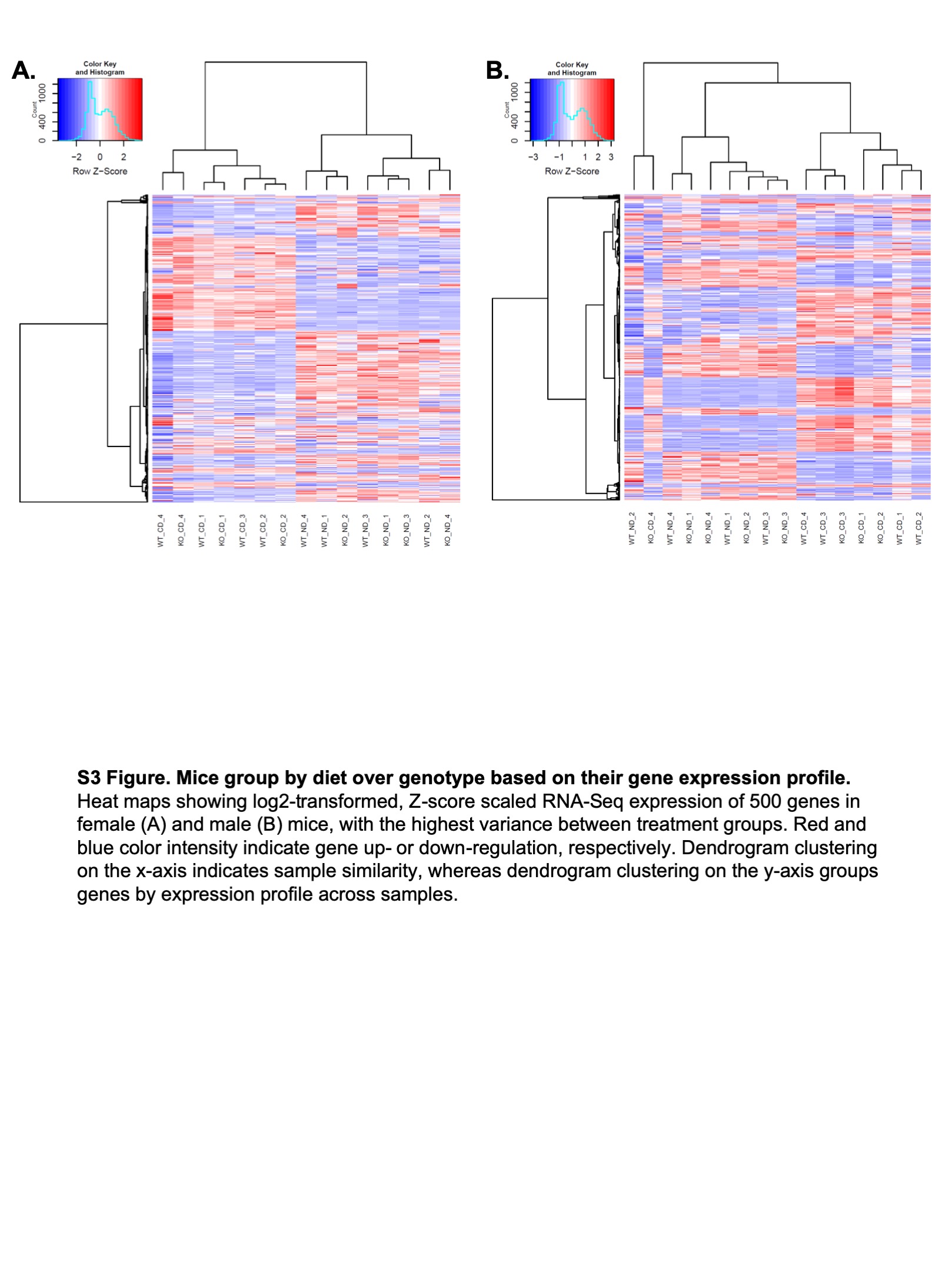

Supplement: S3 Fig — Heat maps showing log2-transformed, Z-score scaled RNA-Seq expression of 500 genes in female (A) and male (B) mice, with the highest variance between treatment groups. Red and blue color intensity indicate gene up- or down-regulation, respectively. Dendrogram clustering on the x-axis indicates sample similarity, whereas dendrogram clustering on the y-axis groups genes by expression profile across samples. (JPEG) [file pone.0229896.s004.jpeg]

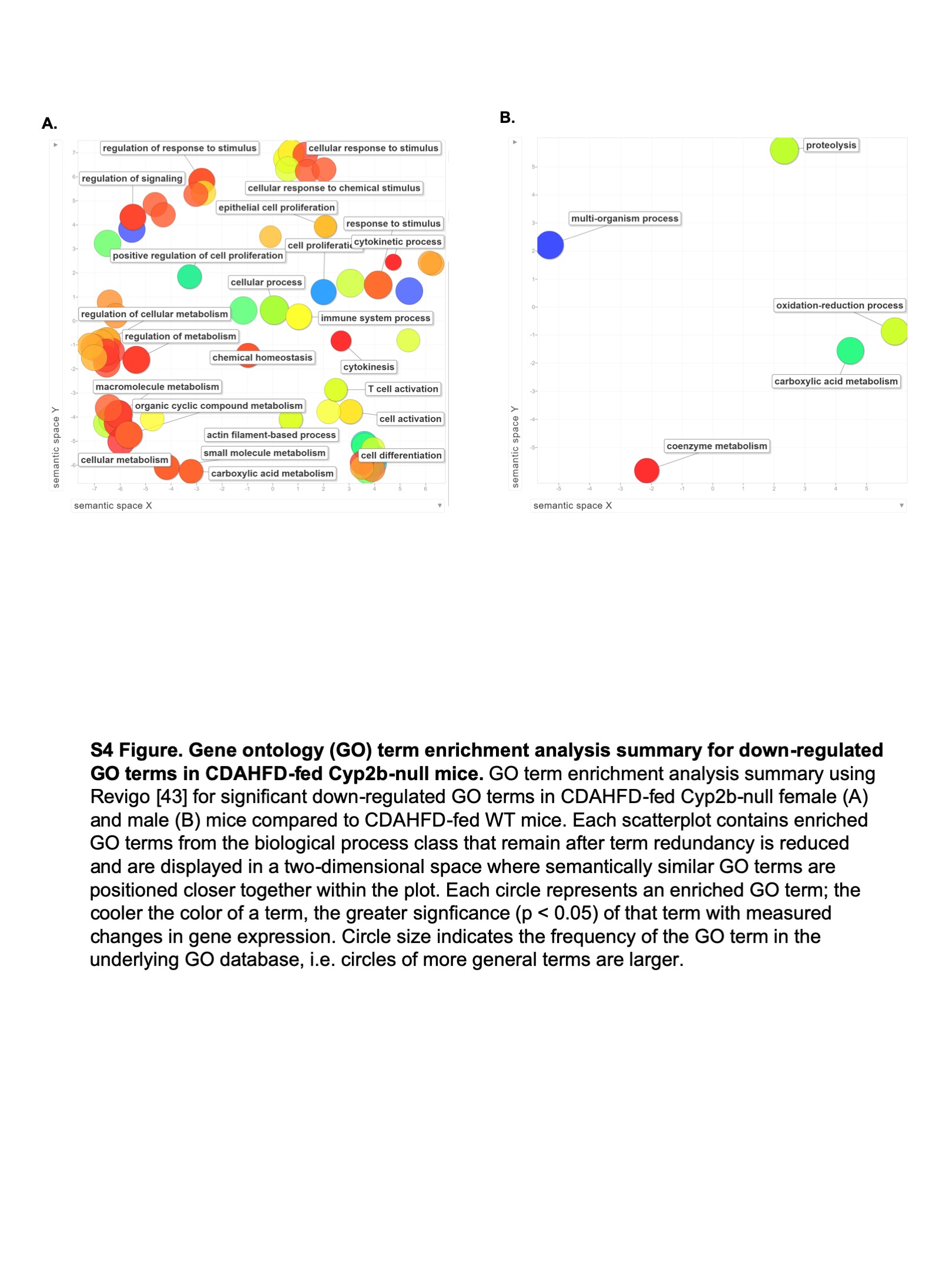

Supplement: S4 Fig — GO term enrichment analysis summary using Revigo [43] for significant down-regulated GO terms in CDAHFD-fed Cyp2b-null female (A) and male (B) mice compared to CDAHFD-fed WT mice. Each scatterplot contains enriched GO terms from the biological process class that remain after term redundancy is reduced and are displayed in a two-dimensional space where semantically similar GO terms are positioned closer together within the plot. Each circle represents an enriched GO term; the cooler the color of a term, the greater signficance (p < 0.05) of that term with measured changes in gene expression. Circle size indicates the frequency of the GO term in the underlying GO database, i.e. circles of more general terms are larger. (JPEG) [file pone.0229896.s005.jpeg]

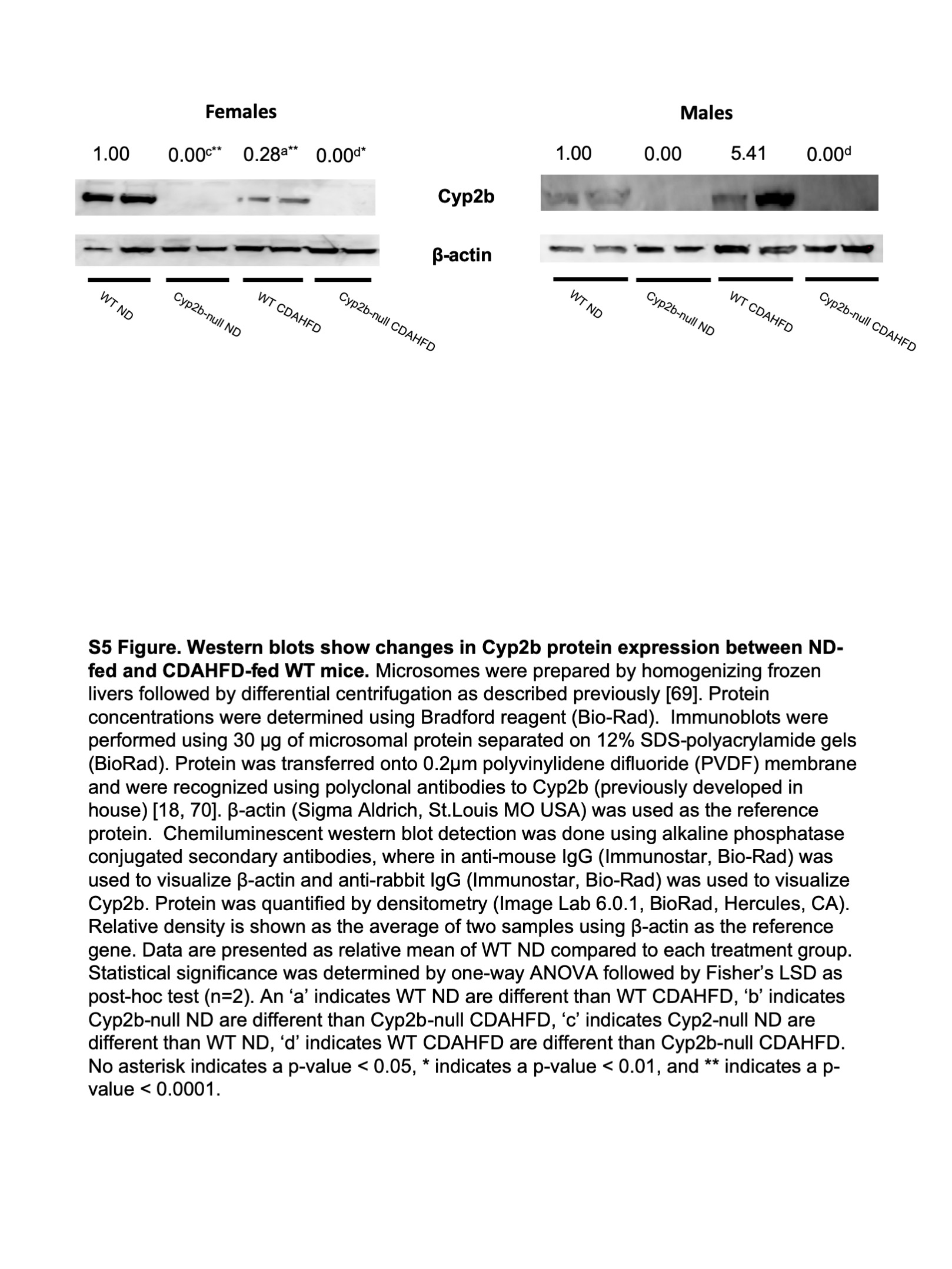

Supplement: S5 Fig — Microsomes were prepared by homogenizing frozen livers followed by differential centrifugation as described previously [75]. Protein concentrations were determined using Bradford reagent (Bio-Rad). Immunoblots were performed using 30 μg of microsomal protein separated on 12% SDS-polyacrylamide gels (BioRad). Protein was transferred onto 0.2μm polyvinylidene difluoride (PVDF) membrane and were recognized using polyclonal antibodies to Cyp2b (previously developed in house) [18, 76]. β-actin (Sigma Aldrich, St. Louis MO USA) was used as the reference protein. Chemiluminescent immunoblot detection was done using alkaline phosphatase conjugated secondary antibodies, where in anti-mouse IgG (Immunostar, Bio-Rad) was used to visualize β-actin and anti-rabbit IgG (Immunostar, Bio-Rad) was used to visualize Cyp2b. Protein was quantified by densitometry (Image Lab 6.0.1, BioRad, Hercules, CA). Relative density is shown as the average of two samples using β-actin as the reference gene. Data are presented as relative mean of WT ND compared to each treatment group. Statistical significance was determined by one-way ANOVA followed by Fisher’s LSD as post-hoc test (n = 2). An ‘a’ indicates WT ND are different than WT CDAHFD, ‘b’ indicates Cyp2b-null ND are different than Cyp2b-null CDAHFD, ‘c’ indicates Cyp2-null ND are different than WT ND, ‘d’ indicates WT CDAHFD are different than Cyp2b-null CDAHFD. No asterisk indicates a p-value < 0.05, * indicates a p-value < 0.01, and ** indicates a p-value < 0.0001. (JPG) [file pone.0229896.s006.jpg]
