# Supplementary material for: Gender differences in diet-induced steatotic disease in Cyp2b-null mice
Source: PLoS One. 2020 Mar 10;15(3):e0229896. doi: 10.1371/journal.pone.0229896 (PMC7064244; doi:10.1371/journal.pone.0229896)
Supplement: S6 Fig — Full immunoblot images of PCNA, CYP2B, and β-actin. (A) CYP2B immunoblot: CYP2B is sexually dimorphic and expressed much higher in females than males [55–57]. (B) Microsomal β-actin as the housekeeping protein. (C) PCNA. (D) Nuclear β-actin as the housekeeping protein. Left hand side of blots are often but not always stained with molecular weight markers. Blot images are from S5 Fig (CYP2B and β-actin) and Fig 1(PCNA and β-actin). (PDF) [file pone.0229896.s007.pdf]

S6 Figure. Full immunoblot and gel images required by PLoS ONE. Full immunoblot images of PCNA, CYP2B, and b-actin. (A) CYP2B immunoblot: CYP2B is sexually dimorphic and expressed much higher in females than males. CYP2A is the band located below CYP2B. (B) Microsomal β-actin as the housekeeping protein. (C) PCNA. (D) Nuclear β-actin as the housekeeping protein. Left hand side of blots are often but not always stained with molecular weight markers. Blot images are from **S5 Figure** (CYP2B and β-actin) and **Figure 1** (PCNA and β-actin).

A

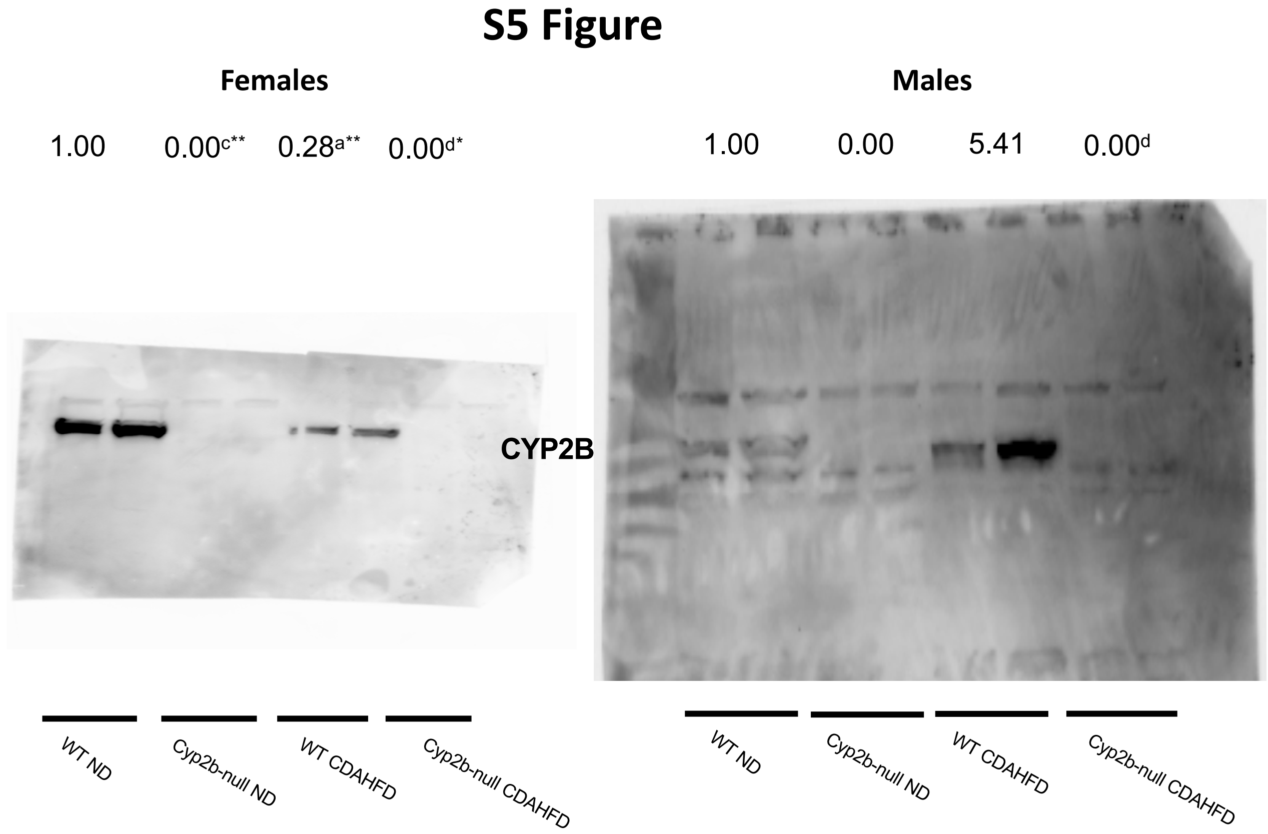

B

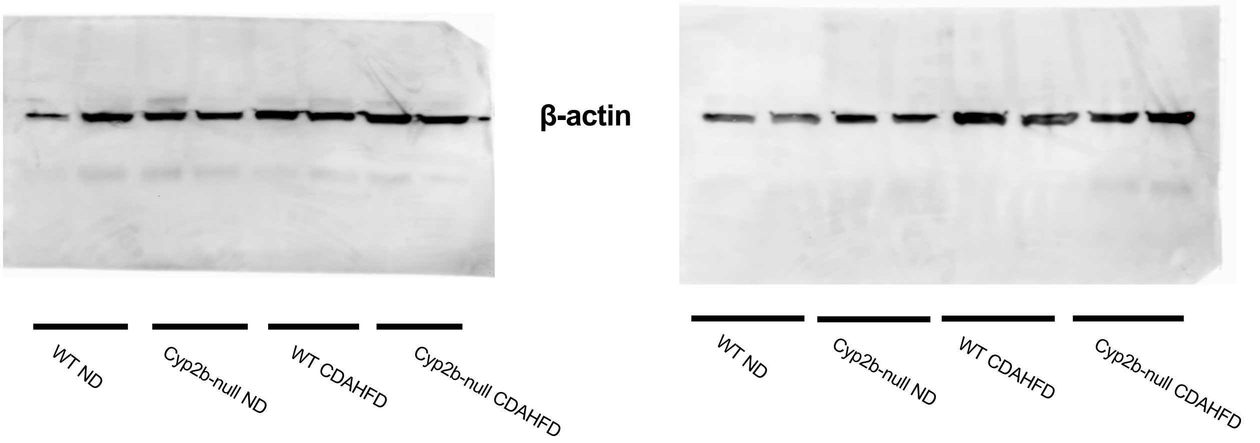

Figure 1

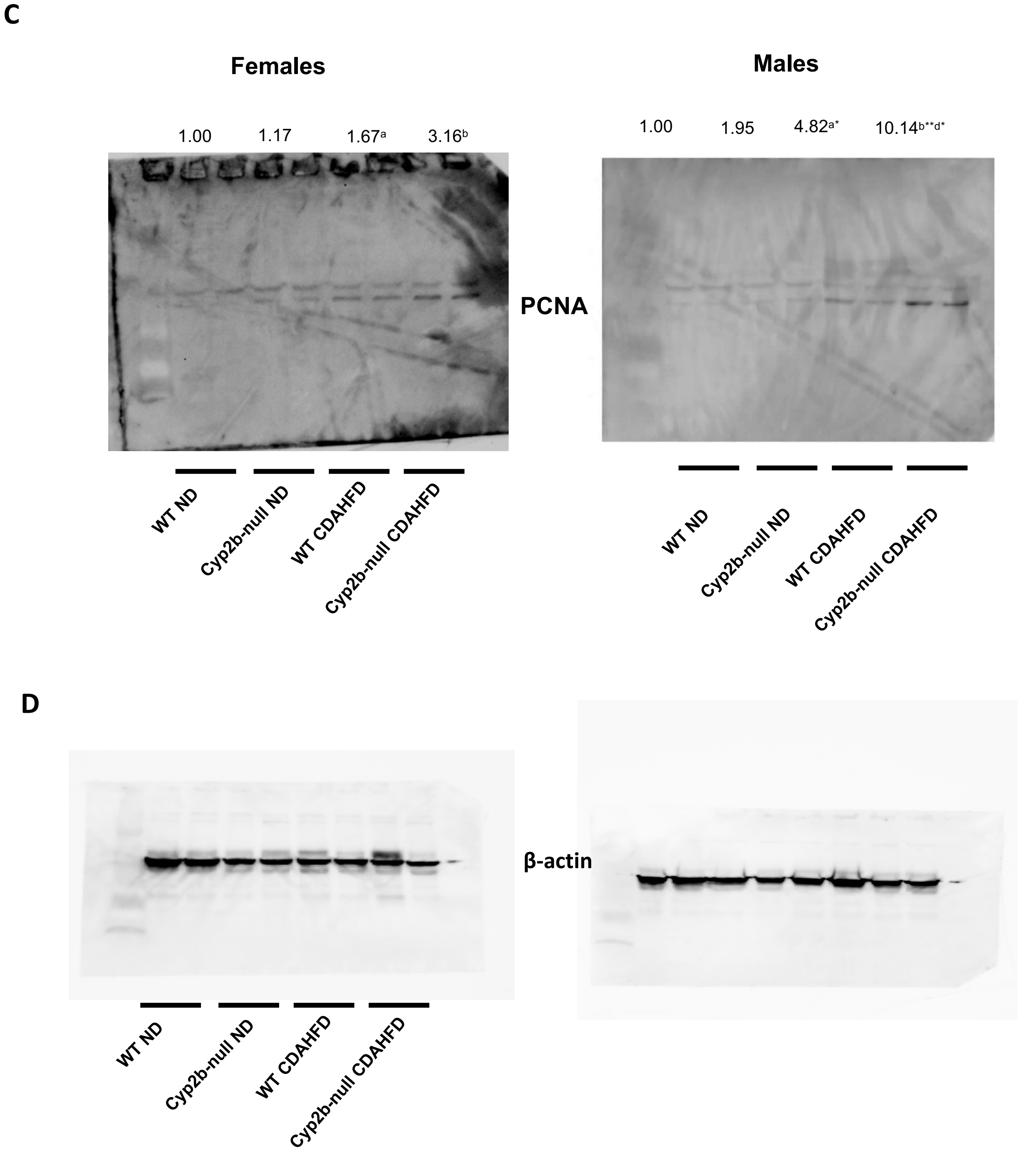

C
Females
1.00
1.17
1.67a
3.16b
PCNA
WT ND
Cyp2b-null ND
WT CDAHFD
Cyp2b-null CDAHFD
Males
1.00
1.95
4.82a*
10.14b**d*
WT ND
Cyp2b-null ND
WT CDAHFD
Cyp2b-null CDAHFD
D
β-actin
WT ND
Cyp2b-null ND
WT CDAHFD
Cyp2b-null CDAHFD
